# Synthesis and Pharmacological Characterization of a Novel Cannabinoid Receptor 1 Antagonist

**DOI:** 10.1021/acsomega.4c11355

**Published:** 2025-06-02

**Authors:** Iker Bengoetxea de Tena, Gorka Pereira-Castelo, Jonatan Martínez-Gardeazabal, Marta Moreno-Rodríguez, Iván Manuel, Claudio Martínez, Belén Vaz, Javier González-Ricarte, Rosana Álvarez, Angel Torres-Mozas, Francesca Peccati, Gonzalo Jiménez-Osés, Angel Rodríguez de Lera, Rafael Rodríguez-Puertas

**Affiliations:** † Department of Pharmacology, Faculty of Medicine and Nursing, 16402 University of the Basque Country (UPV/EHU), Leioa University Campus, Sarriena s/n, 48940, Leioa, Spain; ‡ Neurodegenerative Diseases, BioBizkaia Health Research Institute, Biobizkaia Building, Cruces Square, 48903, Barakaldo, Spain; § CINBIO and Department of Organic Chemistry, Faculty of Chemistry, University of Vigo, Experimental Sciences Building, Lagoas-Marcosende University Campus, 36310, Vigo, Spain; _∥_ 73038 Basque Research and Technology Alliance (BRTA), Bizkaia Technology Park, Building 800, 48160, Derio, Spain; ⊥ Ikerbasque, Basque Foundation for Science, 48013, Bilbao, Spain

## Abstract

The endocannabinoid (eCB) system regulates several brain functions and is implicated in numerous conditions affecting the brain. Thus, the pharmacological blockade of cannabinoid receptors has a therapeutic potential but produces severe psychiatric side effects. Hence, new cannabinoid compounds with different pharmacological profiles are needed to potentially minimize this toxicity. The objective of this study, featuring original chemical insights, pharmacological analysis, and robust computational methods, was to synthesize and characterize a series of novel antagonists/inverse agonists of cannabinoid receptors. To do so, we first synthesized and then screened 11 novel compounds for affinity for cannabinoid receptors. After that, we characterized in depth the pharmacological profile of the most promising one, UVI3502, which showed affinity for two [^3^H]­CP55,940 binding sites (IC_50Hi_ 0.026 ± 0.43 nM and IC_50Lo_ 772 ± 49.40 nM, *R* ^2^ = 0.59) in the rat cortex. Binding assays performed in membranes overexpressing cannabinoid receptors 1 and 2 (CB_1_ and CB_2_) confirmed moderate affinity for both receptor subtypes, about 10-fold higher for the first one, indicating limited receptor subtype specificity. In key brain areas from the rodent brain, which have a much higher CB_1_ receptor density than CB_2_, the affinity of UVI3502 was further studied with neuroanatomical specificity by autoradiography. Functional [^35^S]­GTPγS assays demonstrated that UVI3502 behaved as an antagonist of CB_1_ receptors, blocking the stimulation evoked by the potent cannabinoid receptor agonist CP55,940. The in silico characterization of the binding to the CB_1_ receptor through molecular docking and molecular dynamics suggests that this activity is explained by the planar and rigid structure of UVI3502, which is optimal for interactions with the inactive state of the receptor. Hence, we synthesized and characterized UVI3502 as a novel antagonist of CB_1_, making it a new pharmacological tool for the study of the eCB system and for blocking cannabinoid receptors in the central nervous system.

## Graphical abstract

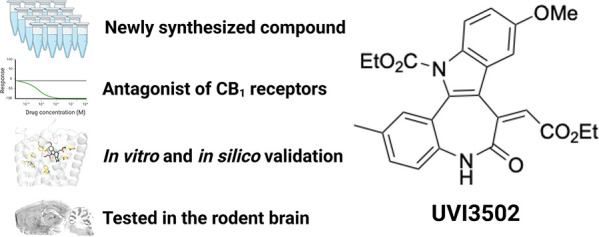

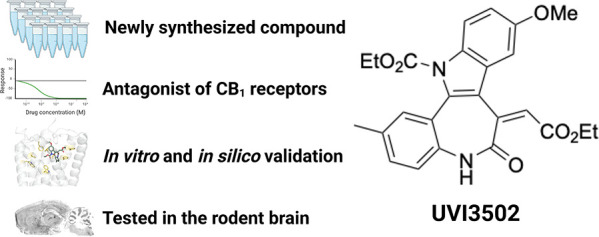

## Introduction

The endocannabinoid (eCB) system is essential for preserving energy balance and metabolism^1^ but is also implicated in modulating cognitive functions, including learning and memory.^2^ This system involves two identified G protein-coupled receptors (GPCRs), cannabinoid receptors of type-1 and -2, or CB_1_ and CB_2_, respectively. Their expression differs significantly, with CB_1_ exhibiting high levels of expression in the central nervous system (CNS), including in brain areas associated with the psychoactive effects of Δ-9-tetrahydrocannabinol (Δ^9^-THC); the basal ganglia, the cerebellum, portions of the hippocampus, and the cortical regions.^3^ In contrast, the areas with the highest levels of CB_2_ receptors are primarily the immune system and the spleen.^4,5^

As one of the most relevant neuromodulatory networks of the CNS, the eCB system regulates important physiological processes, such as neurodevelopment, synaptic plasticity, and adaptive responses,^2^ all of which affect cognition. Hence, despite the well-known deleterious effects of cannabinoids on memory,^6^ there is a growing interest in developing new cannabinoid compounds for the treatment of neurological and neurodegenerative diseases.^7,8^ In fact, various components of the eCB system undergo alterations in post-mortem human samples from Alzheimer’s disease (AD) patients,^9–11^ and animal model studies suggest that the pharmacological manipulation of the eCB system can influence the histopathological and biochemical markers associated with this disease.^12–14^ In addition to AD, the potential of cannabinoid treatments in animal models of other neurodegenerative and also neurodevelopmental disorders, including Parkinson’s (PD) and Huntington’s diseases (HD), or Williams–Beuren syndrome, has also been described.^15–18^

While many of these treatments involve the activation of the eCB system, either through direct action on cannabinoid receptors or through regulation of the synthesis and degradation enzymes of endocannabinoids, the pharmacological blockade of cannabinoid receptors also exerts positive effects depending on the context. For instance, in two mouse models of Down’s syndrome (DS), treatments with the gold-standard inverse agonist of CB_1_ receptors, SR141716A or rimonabant,^19^ restored key cognitive phenotypes affected by the pathology.^20^ Similarly, in another neurodevelopmental condition, fragile X syndrome, CB_1_ blockade restored cognition and normalized the morphology of dendritic spines, while blocking CB_2_ only normalized anxiety levels.^21,22^ In a very different context, mice treated with the well-known muscarinic antagonist scopolamine, known to produce transient cholinergic hypofunction and cognitive deficits, cotreatment with MK-7128, a CB_1_ receptor inverse agonist, improved performance in different behavioral tasks and this was achieved at moderate levels of CB_1_ occupancy in the brain.^23^

The pharmacological inhibition of cannabinoid receptors, particularly CB_1_, offers the potential to ameliorate a wide array of cognitive deficits arising from various causes. However, the most used compound in these studies, rimonabant, is a very high-affinity inverse agonist which produced severe psychiatric side effects, including anxious and depressive disorders, when it was administered to patients suffering from obesity.^24,25^ Thus, there is a need to develop new compounds with a similar pharmacological profile that can potentially avoid such adverse effects.

Thus, our objective was to perform the chemical synthesis and pharmacological characterization in the rodent brain using in vitro and in silico methods of a number of novel antagonists/inverse agonists of cannabinoid receptors. We describe that one of these compounds, UVI3502, is a novel antagonist of CB_1_ receptors, which blocks the stimulation produced by CP55,940, a potent cannabinoid agonist, in some of the most relevant brain areas that control learning and memory processes.

## Results and Discussion

Considering the therapeutic potential of the eCB system regarding various conditions affecting the brain, there is a need to develop novel compounds targeting cannabinoid receptors both as new research tools and as potential treatments for these disorders. In this study, we have performed the synthesis and pharmacological screening of a series of novel compounds for affinity for cannabinoid receptors, followed by a pharmacological profiling of the most promising compound, using a range of in vitro and in silico methods. Hence, this work encompasses the comprehensive process of drug discovery of a novel compound, detailing the design, chemical synthesis, and in vitro and in silico pharmacodynamical characterization.

### Chemical Synthesis of the Novel Compounds

As a follow-up to previously described studies regarding the palladium-catalyzed heterocyclization/oxidative Heck coupling cascade as a synthetic approach to fused heterocycles, we have explored the feasibility of performing the reaction sequence in a one-pot fashion. The merge of Sonogashira heterocyclization and oxidative Heck processes in the same step using the same catalyst generates a new series of dihydroindolo­[3,2-*d*] benzazepine-6­(5H)-ones starting from simple protected ortho-iodoanilines and acyl ortho-alkynylanilines (see Scheme 1).

**Scheme 1:**
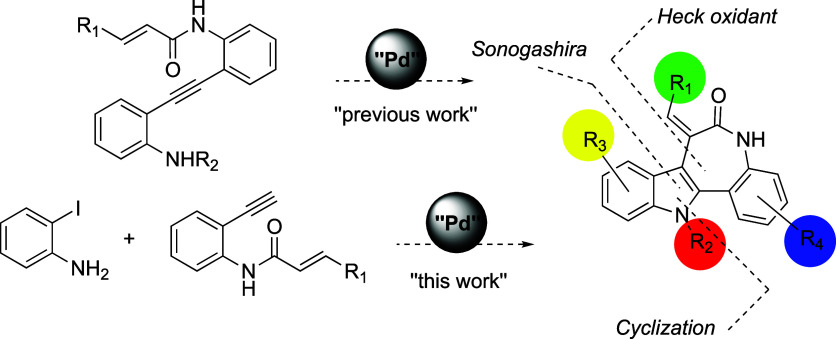
Synthetic Approach to the Construction of the dihydroindolo­[3,2-*d*] Benzazepine-6­(5H)-One Skeleton, including the Intra- and Intermolecular Version

In the synthetic design, we have also considered the better solubility of the indole derivatives protected as carbamates, which are easier to purify and crystallize than the corresponding free indoles with the same substitution pattern, while the carbamate group does not prevent cyclization through the nitrogen in the nucleopalladation step.^26^

The synthesis of the precursors included two straightforward reactions, as shown in Scheme 2, namely, the previously described procedures for 2-haloarylcarbamates **2** ^26,27^ and the condensation of the 2-ethynylanilines **3** with the corresponding acid chloride for the acyl derivatives **4**, both proceeding in good yields.^26,28,29^

**Scheme 2:**
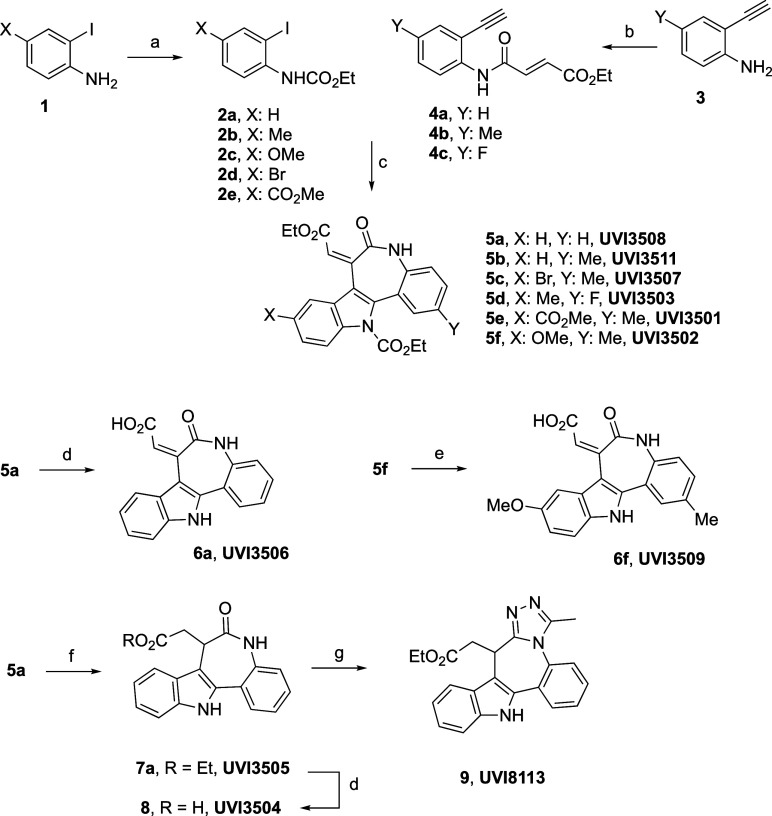
**Reagents and Reaction Conditions**:
a ClCO_2_Et, K_2_CO_3_, Acetone, 25 °C (**2a**–**e**, 74-81%).
b Ethyl Fumaroyl Chloride, NaHCO_3_, Dioxane, 0 to 25 °C, 23 h (**4a**–**c**, 66-90%).
c (i) PdCl_2_(PPh_3_)_2_ (5 mol %), CuI (20 mol %), PPh_3_ (5 mol %), Et_3_N, DMF, 60 °C, 12 h and (ii) Air, 100 °C, 15 h (**5a**–**f**, 47-75%).
d LiOH H_2_O, THF, 50 °C, 12 h (**6a**, 86%; **8**, 79%).
e TBAF, THF, 80 °C, 21 h (**6f**, 71%).
f (i) H_2_, Pd­(OH)_2_, EtOAc, 25 °C and (ii) 1M TBAF in THF, 80 °C, 21 h (**7a**, 73%).
g (i) Lawesson’s Reagent, THF, 60 °C, 3 h; (ii) H_2_NNH_2_·H_2_O, THF, 20 °C, and (iii) CH_3_C­(OEt)_3_, THF, 80 °C, 1 h (**9**, 87%).

Based on the previously reported conditions for the formation of fused indoles in a one-pot sequence,^26^ the construction of the target skeleton included: (a) treatment of the corresponding ortho-iodoaniline **2** (1 equiv) and alkyne **4** (2 equiv) with PdCl_2_(PPh_3_)_2_ (5 mol %) as the catalyst and CuI (20 mol %), Ph_3_P (5 mol %), and Et_3_N (2 equiv) as the additives in *N*,*N*-dimethylformamide (DMF) under an argon atmosphere at 50 °C for 0.5 h and (b) opening the flask to air and heating to 50 °C for 17–22 h. Indoles **5a**–**f** were obtained in 47–75% yields depending upon the substitution pattern, which can be considered as a very efficient protocol given the increase in structural complexity resulting from three consecutive synthetic steps.

Straightforward synthetic modifications (deprotection and hydrolysis of the ester) allowed conversion of the carbamate/ester of **5a** into **6a** and **5f** into **6f** (see Scheme 2). Hydrogenation of the conjugated ester **5a** upon catalysis of Pd­(OH)_2_ in ethyl acetate and deprotection of the carbamate with TBAF afforded **7a** in a combined 73% yield, which was alternatively hydrolyzed to **8** or converted into the fused triazole **9** in an overall 87% yield upon combined treatment with Lawesson’s reagent at 60 °C, followed by hydrazine hydrate and triethyl orthoacetate in THF at 80 °C (see Scheme 2).

With the synthetic approach used, we have further increased the efficiency of the intramolecular oxidative Heck cascade reaction,^26^ by incorporating a Sonogashira cross-coupling prior to the heterocyclization–Heck, as described for other heterocycles. This strategy led to the formation and *N*-cyclization of ortho-alkynylaniline intermediates starting from appropriately protected ortho-iodoanilines and terminal alkynylanilines. The sequence of chemical transformations was performed in the same reaction flask while additional reagents and catalysts were added at different time intervals,^30^ another example of the “one-pot” multicomponent reaction (MCR).^31^ This combination of consecutive Sonogashira, nucleopalladation, and oxidative Heck couplings conveniently allowed the preparation of a new series of 7,12-dihydroindolo­[3,2-*d*]­benzazepine-6­(5H)-ones. The skeleton of the indolobenzazepinones was further modified by incorporation of additional substituents or by its conversion into the fused [1,2,4]­triazoloazepines in an efficient manner.

### Screening of the Newly Synthesized Compounds for Cannabinoid Receptor Affinity

Following the synthesis, we evaluated the pharmacodynamic parameters of the novel compounds using radioligand affinity assays with [^3^H]­CP55,940 performed in membrane homogenates purified from the rat cortical tissue, which naturally contain CB_1_ and CB_2_ receptors.^5^ Rat cortical tissue was used for these assays, given the aforementioned relevance of the eCB system in neurological conditions.

The IC_50_ values for each compound obtained in the competition curves are summarized in Table S1. A comparison of fits was performed for every curve, and the statistically preferred model (one site vs two sites) was chosen in each case. Out of the 11 compounds analyzed, one of them, UVI3502, showed affinity for cannabinoid receptors. UVI3502 showed a relatively high affinity with two binding sites in the [^3^H]­CP55,940 competition curve (IC_50Hi_ 0.026 ± 0.43 nM and IC_50Lo_ 772 ± 49.40 nM, *R* ^2^ = 0.59; see Figure 1 and Table S1). Given that the assay was performed using a tritiated agonist, [^3^H]­CP55,940, the two binding sites observed are expected to correspond to different receptors, most likely CB_1_ or CB_2_, rather than to two different affinity states (e.g., high and low) of the same receptor, which can only be observed by performing the competition with a tritiated antagonist.^32^ The total inhibition of [^3^H]­CP55,940 binding exerted by UVI3502 was about 58%, with approximately 18% corresponding to the high-affinity binding site and the remaining 40% corresponding to the low-affinity one. None of the other compounds showed significant affinity for cannabinoid receptors (i.e., inhibition of [^3^H]­CP55,940 binding). Consequently, UVI3502 was selected as the best candidate compound for subsequent pharmacodynamic characterization using both in vitro and in silico techniques.

**Figure 1:**
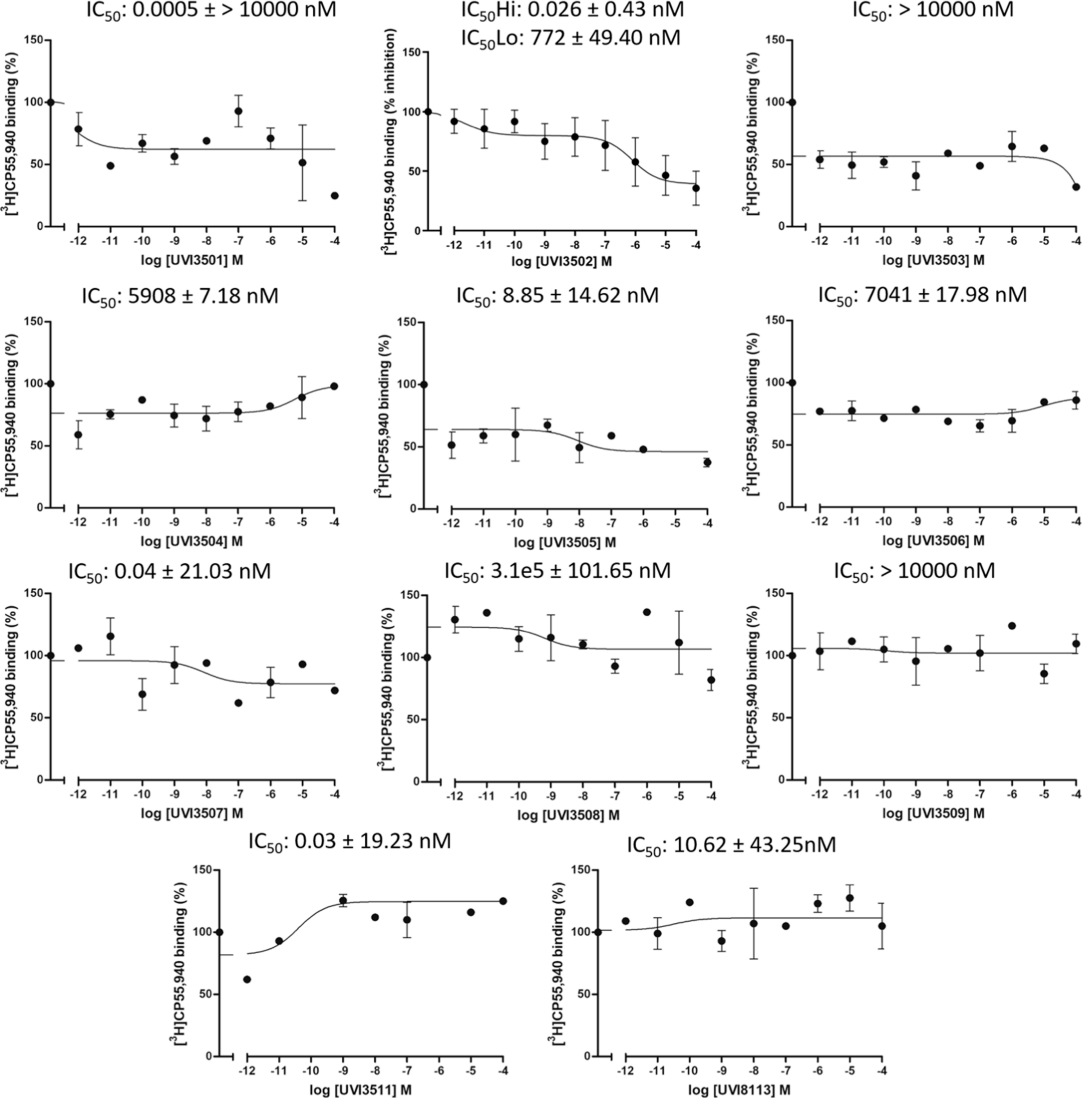
Competition curves of [^3^H]­CP55,940 vs increasing concentrations ranging from 10^–12^ M to 10^–4^ M of the 11 novel compounds in membrane homogenates from the Sprague–Dawley rat brain cortex (*n* = 5). Out of the 11 compounds, one of them, UVI3502 (center up), inhibited [^3^H]­CP55,940 binding with two different binding sites, likely corresponding to two different receptor subtypes. Curves represent the mean of *n* = 2 independent experiments with a range of 4–5 technical replicates for every compound except UVI3502, in which the curve represents the mean of *n* = 5 independent experiments with 11 technical replicates.

### Screening of the Affinity of UVI3502 for Cannabinoid Receptor Subtypes

The pharmacological profiling of UVI3502 as a novel cannabinoid ligand was completed by performing inhibition curves of [^3^H]­CP55,940 vs a range of concentrations of UVI3502 in membrane homogenates from CHO cells overexpressing human CB_1_ and CB_2_ receptors. As a further control, inhibition curves were also performed in the rat spleen tissue, due to the high expression of CB_2_ and very low levels of CB_1_ receptors in this organ.^33^

UVI3502 showed affinity for CB_1_ receptors and a single binding site in CB_1_ overexpressing cells (IC_50_ 4641 ± 1595 nM, *R* ^2^ = 0.55; see Figure 2A), following the same comparison of fits performed (one site vs two sites). The total inhibition of [^3^H]­CP55,940 binding exerted by UVI3502 in this tissue was approximately 38%. These results indicate that UVI3502 partially binds the CB_1_ receptor. UVI3502 also showed approximately 10-fold lower affinity for the CB_2_ receptor, inhibiting [^3^H]­CP55,940 binding in CB_2_ overexpressing cells and in spleen membrane homogenates in concentrations at the low micromolar range (CB_2_ overexpressing cells: IC_50_ 16200 ± 130.67 nM, *R* ^2^ = 0.83; see Figure 2B; spleen membrane homogenates: IC_50_ 10230 ± 17.83 nM, *R* ^2^ = 0.62; see Figure 2C). The maximum inhibition achieved was approximately 83% and 61%, respectively, indicating that UVI3502 partially displaces [^3^H]­CP55,940 binding in cells overexpressing the CB_2_ receptor with low affinity. Together, the results obtained in [^3^H]­CP55,940 binding assays performed in the rat cortical tissue as well as in CB_1_ and CB_2_ overexpressing membrane homogenates indicate a limited receptor subtype specificity of UVI3502 for CB_1_, and the possibility that the high-affinity binding site observed in Figure 1 could correspond to a third, non-CB_1_/-CB_2_, receptor.^34^

**Figure 2:**
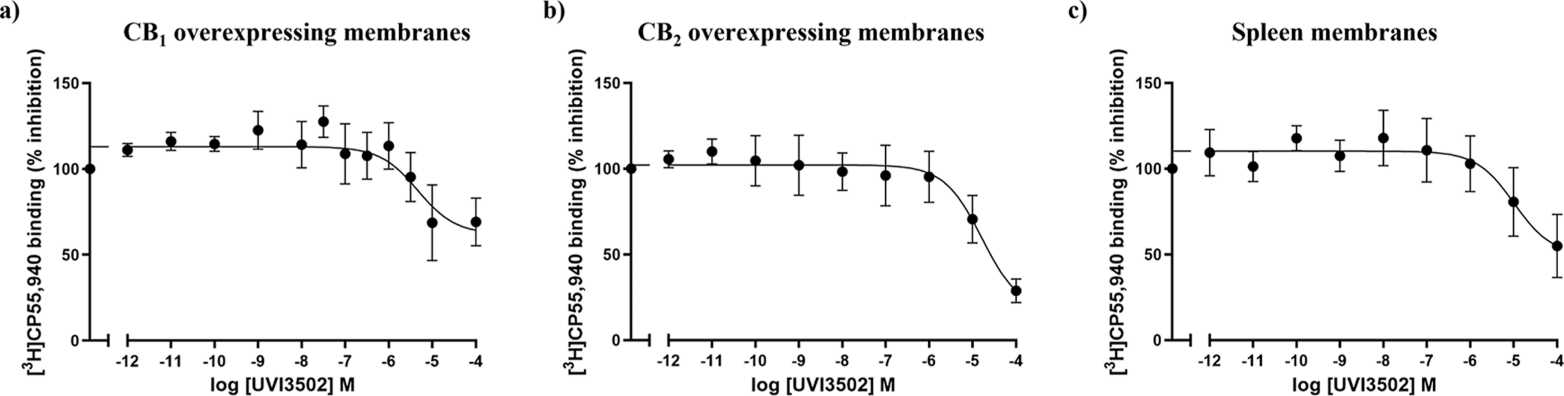
Inhibition curves of [^3^H]­CP55,940 vs increasing concentrations ranging from 10^–12^ M to 10^–4^ M of UVI3502 in (a) CB_1_ overexpressing membranes, (b) CB_2_ overexpressing membranes, and (c) Sprague–Dawley rat spleen membranes (*n* = 5). Note that the curve shows a single binding site with CB_1_ overexpressing membranes, indicating that UVI3502 binds CB_1_ receptors (IC_50_ 4641 ± 1595 nM, *R* ^2^ = 0.55). Note that UVI3502 also shows low affinity for CB_2_ receptors both in CB_2_ overexpressing membranes (IC_50_ 16200 ± 130.67 nM, *R* ^2^ = 0.83) and in CB_2_-enriched spleen tissue (IC_50_ 10230 ± 17.83 nM, *R* ^2^ = 0.62). Each curve represents the mean of *n* = 3–4 independent experiments with a range of 6–10 technical replicates.

### Characterization of the Binding of UVI3502 to the CB_1_ Receptor with Neuroanatomical Specificity in the Rodent Brain

The pharmacological profile of UVI3502 was further characterized in the rodent brain with neuroanatomical specificity by performing [^3^H]­CP55,940 autoradiographic assays in brain slices from two most used animal models in research, naive Sprague–Dawley rats and Swiss mice (see Figure 3), focusing on brain regions associated with learning and memory processes.

**Figure 3:**
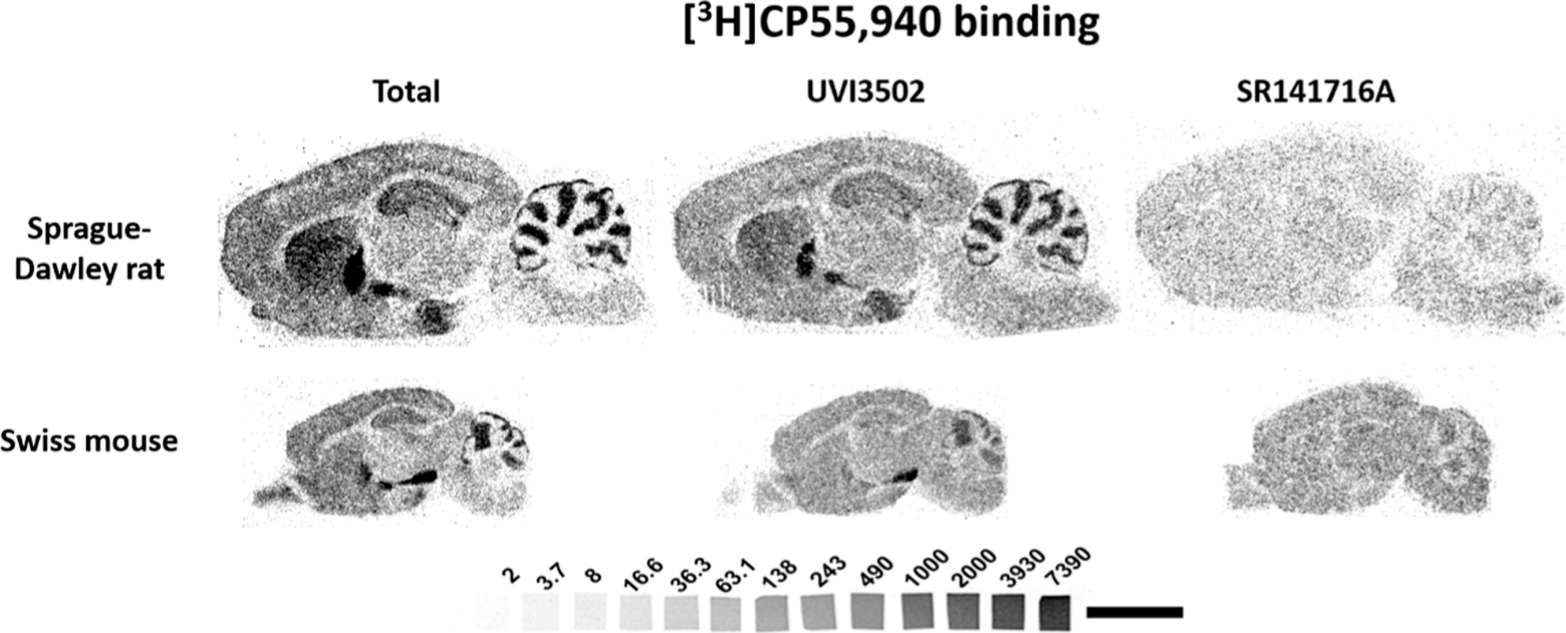
Representative autoradiograms of rat (*n* = 5) and mouse (*n* = 5) brain sagittal sections showing [^3^H]­CP55,940 binding alone, [^3^H]­CP55,940 binding in the presence of UVI3502, and [^3^H]­CP55,940 binding in the presence of SR141716A. [^3^H] microscales used as standards in Ci/g t.e. Note the partial inhibition of [^3^H]­CP55,940 binding by UVI3502 in most of the brain areas expressing CB_1_ receptors. Scale bar = 0.5 cm.

[^3^H]­CP55,940 autoradiography was performed in the presence of both radioligand and UVI3502, as well as in the presence of the radioligand and SR141716A, a known inverse agonist of CB_1_ receptors, in order to have a reference of the amount of [^3^H]­CP55,940 binding inhibited by the novel compound. UVI3502 was able to partially inhibit [^3^H]­CP55,940 binding in all areas that were analyzed in both rat and mouse brain slices (see Table S2). Given the much higher density of CB_1_ over CB_2_ receptors in the brain,^35,36^ these results confirm that UVI3502 binds CB_1_ receptors and indicate that it inhibits a fraction of the [^3^H]­CP55,940 binding inhibited by the full inverse agonist SR141716A.

### [^35^S]­GTPγS Functional Assay to Characterize the Activity of UVI3502 at the CB_1_ Receptor

To study the activity of UVI3502 at CB_1_ receptors, [^35^S]­GTPγS functional assays in membrane homogenates from the rat cortical tissue and in CHO cells overexpressing the human CB_1_ receptor were performed. In these assays, UVI3502 did not stimulate the coupling of CB_1_ receptors to G_i/o_ proteins in any of the tissues used, and no significant reductions in baseline levels of G_i/o_ protein coupling were observed, especially in CB_1_ overexpressing cells. These results suggest that UVI3502 acts as an antagonist of CB_1_ receptors (see Figure S1).

### Characterization of the Activity of UVI3502 with Neuroanatomical Specificity in the Rodent Brain

A subsequent functional autoradiographic assay to determine the activity of UVI3502 in key brain areas of the rodent brain was then conducted. The assay was performed by incubating [^35^S]­GTPγS with CP55,940 (10 μM) alone, as a known agonist of CB_1_ receptors, and consecutive slices with UVI3502 (10 μM) alone and in the presence of both CP55,940 (10 μM) and UVI3502 (10 μM) (see Figure 4).

**Figure 4:**
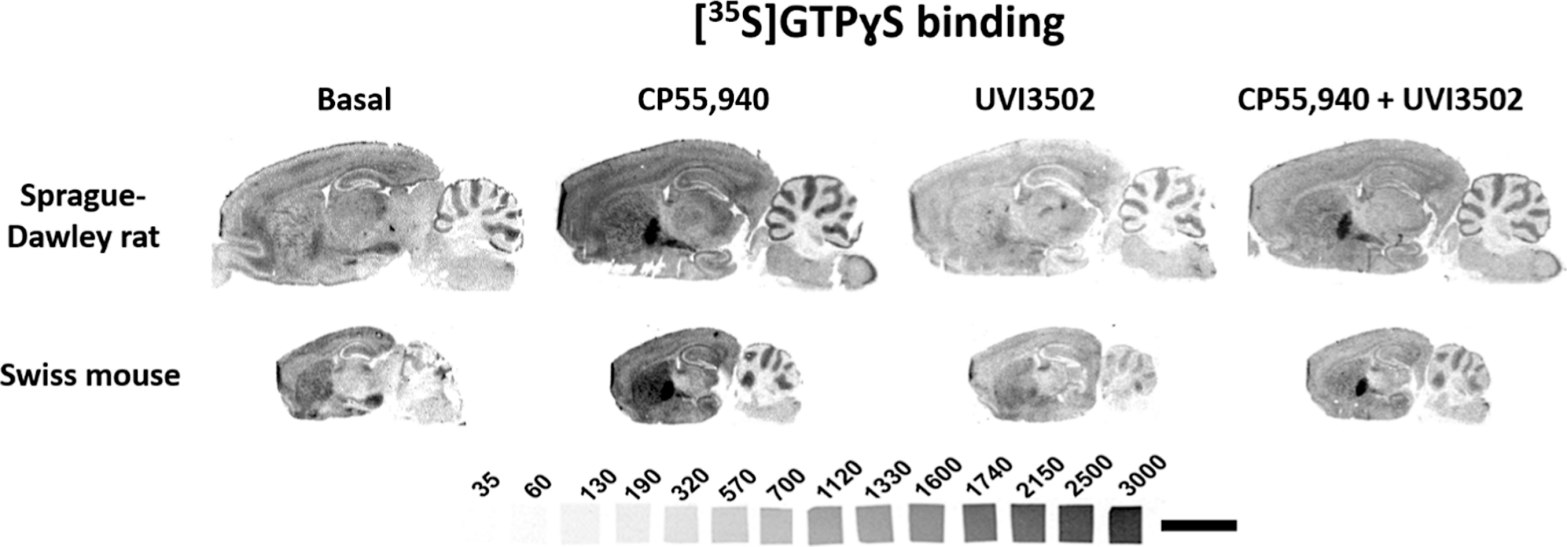
Representative autoradiograms of rat (*n* = 5) and mouse (*n* = 5) brain sagittal sections that show [^35^S]­GTPγS basal binding as well as [^35^S]­GTPγS binding in the presence of CP55,940 (10 μM) alone, in the presence of UVI3502 (10 μM) alone and in the presence of both CP55,940 and UVI3502 (both at 10 μM). Note that UVI3502 inhibits the stimulation evoked by CP55,940 in most brain areas, indicating that UVI3502 behaves as a CB_1_ antagonist. [^14^C] microscales used as standards in Ci/g t.e. Scale bar = 0.5 cm.

Results from Sprague–Dawley rat and Swiss mouse brain slices indicate that UVI3502 acts as an antagonist, as the stimulation evoked by the agonist CP55,940 was suppressed in all of the analyzed areas (see Table S3). In spite of the fact that UVI3502 only inhibits a fraction of [^3^H]­CP55,940 binding (see Figure 3 and Table S2), it acts as a potent antagonist in suppressing CP55,940-evoked G-protein coupling (see Figure 4 and Table S3), and this highlights its potential as a research tool for the study of the eCB system in the brain.

Interestingly, and unlike what was observed in the [^35^S]­GTPγS functional assays in rat cortex membrane homogenates and in CHO cells overexpressing the human CB_1_ receptor, [^35^S]­GTPγS binding in the presence of UVI3502 was lower than baseline levels of [^35^S]­GTPγS binding in the amygdala, the cortex, the hippocampus, the nucleus basalis magnocellularis (NBM), the striatum, and the gray matter of the cerebellum (see Table S3). These results could indicate an inverse-agonist-like activity of UVI3502 in these areas. In functional autoradiography, the interpretation of basal [^35^S]­GTPγS binding in the presence of no drug, and thus of the concept of “inverse agonist”, remains a subject of controversy. While numerous reports suggest that different receptors can be constitutively active in the absence of any ligand,^37–39^ other reports suggest roles of endogenous ligands in so-called basal activity, such as the formation of adenosine during incubation^40^ or the presence of endogenous lysophosphatidic acid (LPA) activating LPA_1_ receptors.^41^ The seemingly contradictory data in [^35^S]­GTPγS assays performed in membrane homogenates vs brain autoradiography might derive from the different protocols used in both cases and could be affected by the higher presence of endogenous ligands in the brain tissue.

The activity of UVI3502 as an antagonist is noteworthy, given that it shares some structural resemblance to the aminoalkylindole WIN55,212–2, a potent cannabinoid receptor agonist.^42^ To explain this counterintuitive observation, molecular docking and classical molecular dynamics were performed, modeling the binding of UVI3502 to the human CB_1_ receptor.

### Modeling of UVI3502 Binding to CB_1_

Binding of UVI3502 to the human CB_1_ receptor was modeled through a combination of molecular docking and classical molecular dynamics. UVI3502 and related derivatives were docked on a three-dimensional model of CB_1_ generated from its crystallographic structure in complex with the known antagonist/inverse agonist AM-6538 (see Figure 5A).^43^ Figure 5B shows the best scoring docking pose (score = 35.5); like AM-6538, UVI3502 is deeply buried in the binding pocket of CB_1_, roughly occupying the same region, and is engaged in hydrophobic contacts with Phe170, Phe174, Phe268, Trp356, and Phe379. The binding pose is driven by a tight shape complementarity (see Figure 5C); the main differences observed between the two ligands are a reduced occupation of the long channel (lined by Trp279 and Met363) for UVI3502 owing to the size of the carbamate moiety and increased interactions with the upper part of the pocket (Phe268 and Phe379) through the tricyclic core and methoxy group. The two ligands fit the gap and side pocket regions to a similar extent. Except for UVI3501 featuring a larger ester group at the indole ring, all other analogues show a binding pose similar to UVI3502 with the same arrangement of the aromatic core inside the binding site. Consequently, the flexible carbamate, carboxyl, or ester moieties extend into the long channel, and the carboxylic or ester groups occupy the gap region (see Figure S2). Compounds equipped with the rigid tricyclic hydrophobic core (vs tetracyclic ones) and an ester group (vs a carboxylate) show the best binding properties to the CB_1_ receptor. A 100 ns classical molecular dynamics simulation of the UVI3502:CB_1_ complex was performed by using the docking pose as the starting geometry to evaluate the persistence of key binding contacts. Figure 5D shows an overlay of five frames sampled with an even stride from the molecular dynamics simulation. Although some flexibility is observed, the positioning and orientation of UVI3502 in the binding pocket remain constant, as well as the packing of the surrounding hydrophobic residues, corroborating the docking pose as a plausible representation of the binding interaction.

**Figure 5:**
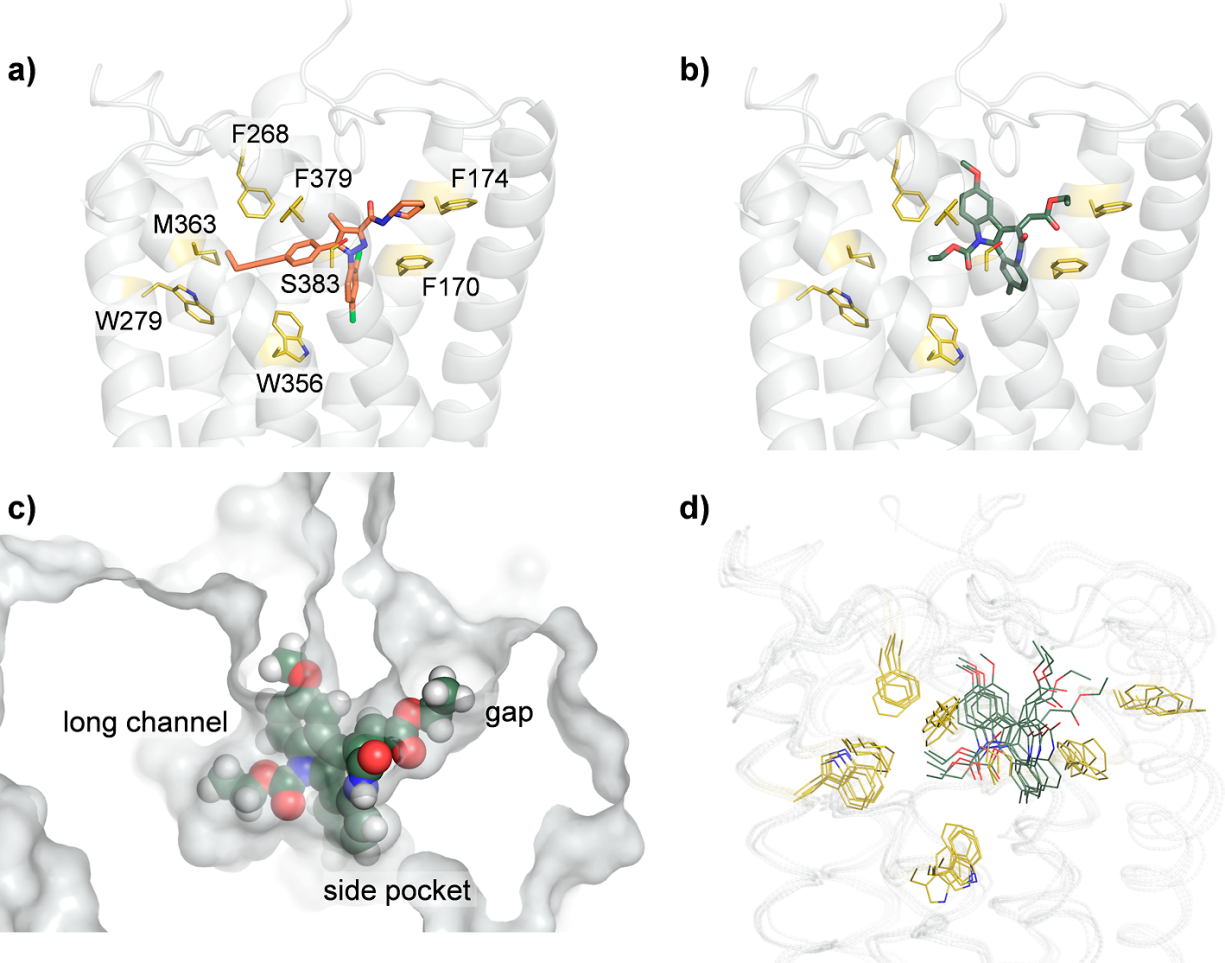
(a) Crystal structure of the human cannabinoid receptor CB_1_ in complex with inverse agonist AM-6538 (in orange sticks; terminal nitrate group is not shown due to the absence of electron density), PDB 5TGZ. (b) Docking pose of UVI3502 (in green sticks) on the human cannabinoid receptor CB_1_ (receptor structure taken from PDB 5TGZ). (c) Space-filling view of the same docking pose with indication of the main regions involved in UVI3502 binding. (d) Overlay of 5 snapshots sampled with an even stride of 20 ns from a molecular dynamics simulation of the UVI3502:CB_1_ complex. Key residues for antagonist/inverse agonist binding are shown as yellow sticks.

The antagonist activity of UVI3502 can be tentatively explained in terms of the shape complementarity with the binding pocket in the active and inactive states of the receptor (Figure 6). Indeed, UVI3502 is predicted to bind in a similar arrangement to previously reported antagonist/inverse agonist AM-6538^43^, extending into the side pocket through its vertical axis and joining the long channel and gap region along its longitudinal axis. The latter is a characteristic feature shared by other known inverse agonists (e.g., AM-251, rimonabant)^44^ and is key for optimal interactions with the receptor in the inactive state (see Figure 6), in which it matches the roughly T-shaped binding cavity. For UVI3502, this is enabled by the quite planar and rigid framework extending from the carbamate to the exocyclic double bond. Conversely, known agonists, such as AM-11542^43^ and WIN55,212–2, bind with an angular shape often emerging from flexible alkyl and aromatic side chains (see Figure 6), which in turn provide optimal contacts with a matching roughly V-shaped cavity in the less longitudinally extended active state. Owing to its rigid framework, we hypothesize that UVI3502 cannot adopt such an angular shape, providing a molecular basis for its activity and providing insights into the structural nuances of CB_1_ activation.

**Figure 6:**
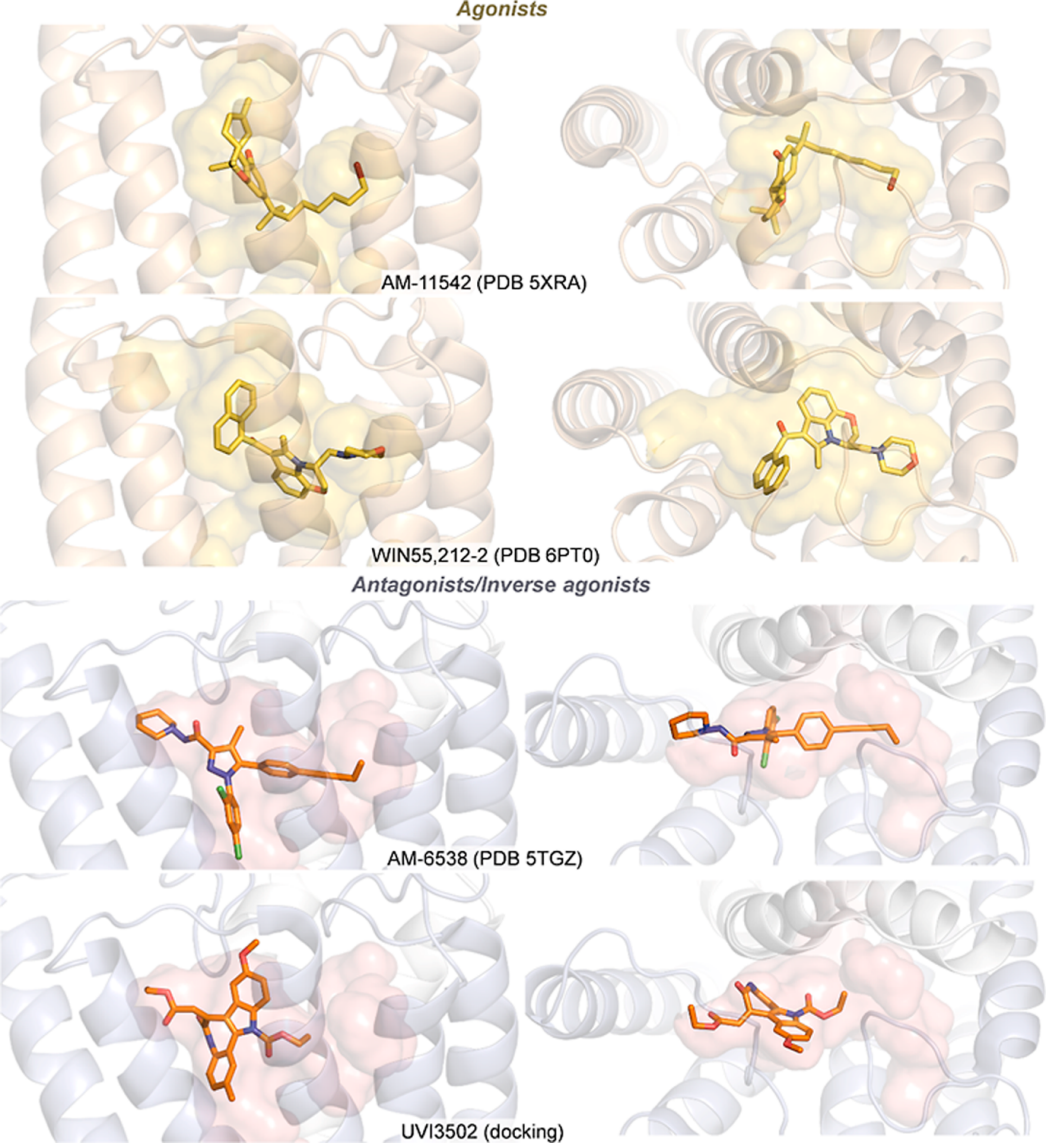
Side view (left) and top view (right) of agonists and antagonists/inverse agonists bound to human CB_1_ (AM-11542, PDB 5XRA; AM-6538, PDB 5TGZ) and CB_2_ (WIN55,212–2, PDB 6PT0) receptors from crystallographic structures and docking pose for UVI3502. The binding cavity is shown as transparent surfaces. Agonists are shown in yellow (top) and antagonists/inverse agonists in orange (bottom).

## Conclusions

In summary, we determined the antagonist properties of the novel compound UVI3502 mainly to CB_1_ receptors, with a 10-fold lower affinity for CB_2_, using both in vitro and in silico approaches. Via functional autoradiography, we determined that UVI3502 efficiently blocks the coupling of CB_1_ to G_i/o_ proteins elicited by a potent cannabinoid agonist in key brain areas controlling learning and memory in the brain tissue from two of the most used experimental models, rats and mice. By using molecular docking and dynamics, we could explain this activity by the planar and rigid structures of UVI3502, which optimally interact with the inactive state of the receptor. While this study offers important insights into the pharmacological properties of UVI3502, it must be acknowledged that the in vitro and in silico methodologies, while robust, may not fully replicate in vivo conditions. The administration of UVI3502 to rodents on its own, as well as by coadministration of this compound along with a potent cannabinoid receptor agonist, like CP55,940, as performed in vitro, would offer valuable information regarding the potential of this compound in therapy. It would be of particular interest to analyze UVI3502 in terms of its effect for the regulation of metabolism, as well as regarding potential psychiatric side effects, which have been observed for other inverse agonists of CB_1_ receptors, most notably rimonabant.^45^ It would also be relevant to analyze the physicochemical properties and in vitro absorption, distribution, metabolism, and excretion (ADME) properties of the compound, such as metabolic stability, intestinal absorption, binding to plasma proteins, or blood–brain barrier permeation. However, these thorough analyses are beyond the scope of the present study and should be investigated in the future. Nevertheless, the present results open the door for the use of this newly synthesized compound as a new research tool for the study of the eCB system and, potentially, for the in vivo inhibition of cannabinoid receptors in the CNS.

## Methods

### Reagents, Drugs, and Chemicals

All necessary compounds for the different procedures were of the highest commercially available quality for the purpose of our studies. For the synthesis of the novel compounds, chemical reagents of the highest purity available were purchased from Sigma-Aldrich and used as received except when indicated.

[^3^H]­CP55,940 (149 Ci/mmol) and [^35^S]­GTPγS (1250 Ci/mmol) were acquired from Revvity (Waltham, MA, USA). The [^3^H]-microscales and [^14^C]-microscales used as standards in the autoradiographic experiments were purchased from ARC (American Radiolabeled Chemicals, St. Louis, MO, USA). The β-radiation sensitive films, Kodak Biomax MR, bovine serum albumin (BSA), DL-dithiothreitol (DTT), guanosine 5′-diphosphate (GDP), guanosine 5′-*O*-3-thiotriphosphate (GTPγS), ketamine, and xylazine were all acquired from Sigma-Aldrich (St Louis, MO, USA).

5-(4-Chlorophenyl)-1-(2,4-dichlorophenyl)-4-methyl-*N*-1-piperidinyl-1H-pyrazole-3-carboxamide hydrochloride (SR141716A) and (11R)-2-Methyl-11-[(morpholin-4-yl)­methyl]-3-(naphthalene-1-carbonyl)-9-oxa-1-azatricyclo­[6.3.1.04,12]­dodeca-2,4(12),5,7-tetraene (WIN55,212–2) were acquired from Tocris (Bristol, UK). (−)-cis-3-[2-Hydroxy-4-(1,1-dimethylheptyl)­phenyl]-trans-4-(3-hydroxypropyl) cyclohexanol (CP55,940) was acquired from Sigma-Aldrich (St Louis, MO, USA). [(1S,2S,5S)-2-[2,6-Dimethoxy-4-(2-methyloctan-2-yl)­phenyl]-7,7-dimethyl-4-bicyclo[3.1.1]­hept-3-enyl]­methanol (HU308) was acquired from Merck (Darmstadt, Germany).

### Chemical Synthesis of the Novel Compounds

We have previously described the palladium-catalyzed heterocyclization/oxidative Heck coupling cascade as a synthetic approach to fused heterocycles, including 3-alkenyl-substituted benzofurans, indoles, 1H-isochromen-1-imines, tetrahydrodibenzofurans, and tetrahydrobenzo­[*c*]­chromen-6-imines^46–50^ and extended the procedure to the synthesis of analogues with the 7,12-dihydroindolo­[3,2-*d*]­benzazepine-6­(5H)-one skeleton. The synthetic protocol allowed the regioselective construction of the core indole and benzazepinone heterocycles of polycyclic compounds, also known as alkylidenepaullones, which were further characterized as activators of the epigenetic enzyme NAD^+^-dependent class of histone deacetylases (sirtuins, Sirt1) in biochemical assays.^26^ The complete information regarding the synthesis of each of the novel compounds is described in detail in the Supporting Information.

### Animals, Tissues and Cells

Animal suffering was minimized to the maximum extent, and the lowest possible number of animals was used. All procedures using all animal species were performed in accordance with the Guide for the Care and Use of Laboratory Animals as adopted and promulgated by the U.S. National Institutes of Health, with the European animal research laws (Directive 2010/63/EU) and the Spanish National protocols, and were approved by the Local Ethical Committee for Animal Research of the University of the Basque Country (CEEA-UPV/EHU 2024/23). All animals used in this study were provided by the general facilities of the University of the Basque Country (UPV/EHU).

#### Sprague–Dawley Rats

Male Sprague–Dawley rats, with a weight of about 200–300 g, were housed in groups of 3–4 per cage, with a constant temperature of approximately 22 °C, in a room with controlled humidity (65%) and with a light/dark cycle of 12:12 h. Animals had access to food and water ad libitum. Spleens and brains from Sprague–Dawley (*n* = 5) rats were used to prepare membrane homogenates for radioligand affinity assays. Brains from Sprague–Dawley rats (*n* = 5) were also used for autoradiographic studies.

#### Swiss Mice

Male Swiss mice, with a weight of about 20–30 g, were housed in a single cage with a constant temperature of approximately 22 °C in a room with controlled humidity (65%) and with a light/dark cycle of 12:12 h. Animals had access to food and water ad libitum. Brains from control Swiss mice (*n* = 5) were used for autoradiographic studies.

#### Membrane Homogenates from CHO Cells Overexpressing CB_1_ and CB_2_ Receptors

Membrane homogenates from CHO cells overexpressing human CB_1_ and CB_2_ receptors, as well as matched wild-type cells, were used to test newly synthesized compounds and were acquired from Sigma-Aldrich (St Louis, MO, USA).

#### Preparation of Membrane Homogenates

Sprague–Dawley rats (*n* = 5) were anesthetized and sacrificed by decapitation. Spleens and brains were then quickly removed by dissection at 4 °C, and in the case of the brain tissue, the cortex was dissected for the preparation of the membrane homogenates. For this procedure, spleen and cortex samples were homogenized using a Teflon-glass grinder (15 up-and-down strokes at 800 rpm) in 30 volumes of homogenization buffer (1 mM EGTA, 3 mM MgCl_2_, 50 mM Tris–HCl; pH 7.4) supplemented with 0.25 mM sucrose, at 4 °C. The obtained homogenates were centrifuged for 5 min at 1500 rpm. Pellets were removed, and supernatants were centrifuged again for 15 min at 14,000 rpm. For washing, the obtained pellets were resuspended in a buffer and centrifuged again, and the supernatant was removed. The resulting aliquots were stored at −80 °C until use.

### Radioligand Binding Assays

#### [^3^H]­CP55,940 Binding Assays

To screen the affinity of the newly synthesized compounds for cannabinoid receptors, these were used in concentrations ranging from 10^–12^ to 10^–4^ M and incubated with a protein concentration of 0.1 mg/mL of rat cortex homogenates (2 h, 37 °C) with agitation. The incubation was performed with 0.5 nM of [^3^H]­CP55,940. To define nonspecific binding, 10^–4^ M of SR141716A was added to the incubation. To stop the reaction, an ice-cold wash buffer (50 mM Tris–HCl and 0.5% BSA, pH 7.4) was added. Then, the membranes were retained by vacuum filtration to a Whatman GF/C glass microfiber filter (Sigma-Aldrich, St. Louis, MO, USA) and the free radioligand was discarded. Filters with the bound radioligand were transferred to vials containing 5 mL of Ultima Gold cocktail (Revvity, Boston, MA, USA) and measured with a Packard Tri-Carb 2200CA liquid scintillation counter (Revvity, Boston, MA, USA).

After that, the compound with the best affinity was tested in cell membrane homogenates overexpressing human CB_1_ and CB_2_ receptors. A concentration of 0.02 mg/mL of commercial WT (as control) and CB_1_ and CB_2_ overexpressing CHO cells were used, and the same protocol described above was followed. Rat spleen homogenates were also used at a concentration of 0.1 mg/mL as a further characterization of binding to the CB_2_ receptor, given the high expression of this receptor in this tissue and the practical lack of CB_1_ in it.^33^ To define nonspecific binding, WIN55,212–2 (CB_1_/CB_2_ agonist) or SR141716A (specific CB_1_ inverse agonist) was added to the incubation, depending on the tissue used for each assay.

#### [^3^H]­CP55,940 Receptor Autoradiography

For the performance of cannabinoid receptor autoradiography using [^3^H]­CP55,940, fresh frozen sections from brain samples from wild-type Sprague–Dawley rats (*n* = 5) and Swiss mice (*n* = 5) were used to test the newly synthesized compound, which had shown the best affinity for cannabinoid receptors.

All brain sections were air-dried for 30 min and later immersed in Coplin jars for preincubation in a buffer containing 50 mM Tris–HCl and 1% of BSA (pH 7.4) for 30 min at room temperature. The objective of this preincubation was to remove endogenous ligands. Two tissue slices were later incubated in the presence of the [^3^H]­CP55,940 radioligand (3 nM) for 2 h at 37 °C and, in two consecutive slices, the incubation was performed also in the presence of the target compound (10 μM) and in the presence of the known CB_1_ inverse agonist SR141716A (10 μM). Following incubation, tissue slices were washed with an ice-cold preincubation buffer, dipped in distilled water, and dried overnight. To generate autoradiograms, dry sections were placed in hermetically closed cassettes and exposed to β-radiation-sensitive films for 21 days at 4 °C. To calibrate the optical densities to fmol/mg tissue equivalent, [^3^H]-microscales were exposed to the films. To quantify the calibrated films after scanning, Fiji software (Bethesda, MA, USA) was used.

#### [^35^S]­GTPγS Functional Binding Assays

The compound with the best affinity was also tested using functional [^35^S]­GTPγS binding assays to characterize its activity as an agonist or antagonist/inverse agonist. A protein concentration of 0.1 mg/mL of rat cortex homogenates and a protein concentration of 0.02 mg/mL of commercial CB_1_ overexpressing CHO cells were used for these assays, suspended in a reaction buffer (Tris–HCl 50 mM, EGTA 1 mM, MgCl_2_ 3 mM, NaCl 100 mM, 0.5% BSA; pH 7.4). The target compound was used in concentrations ranging from 10^–11^ to 10^–4^ M and incubated for 2 h at 37 °C with agitation in the presence of 0.5 nM [^35^S]­GTPγS and 50 μM GDP. Basal coupling of [^35^S]­GTPγS to G_i/o_ proteins was determined by incubating the membrane aliquots with the radioligand in the absence of the target compound. To define nonspecific binding, 10 μM of unlabeled GTPγS was added to the incubation. After the incubation, the same procedure detailed for the [^3^H]­CP55,940 binding assay was followed.

#### Functional [^35^S]­GTPγS Autoradiography

To perform functional autoradiography^51^ of cannabinoid receptors, fresh frozen sections from brain samples from wild-type Sprague–Dawley rats (*n* = 5) and Swiss mice (*n* = 5) were used to test the newly synthesized compound which had shown the best affinity for cannabinoid receptors.

All brain sections were air-dried for 30 min and then immersed in Coplin jars for preincubation (4 times, 15 min each time) in an HEPES-based buffer (50 mM HEPES, 100 mM NaCl, 3 mM MgCl_2_, 0.2 mM EGTA, 0.5% BSA; pH 7.4) at 30 °C. The objective of this preincubation was the removal of endogenous ligands. Slices were then incubated for 2 h at 30 °C in the same buffer supplemented with 2 mM GDP, 1 mM DTT, and 0.04 nM [^35^S]­GTPγS and the target compound (10 μM) alone, as well as the target compound and CP55,940 (10 μM) together. Basal [^35^S]­GTPγS binding was defined in the absence of the agonists in two consecutive slices. To define nonspecific binding, 10 μM of unlabeled GTPγS was added to the incubation in another section. Following incubation, slices were twice washed in an ice-cold 50 mM HEPES buffer (pH 7.4), dried, and exposed for 48 h to β-radiation sensitive film with a set of [^14^C] standards calibrated for [^35^S]. Calibrated films were scanned and quantified using Fiji software (Fiji, Bethesda, MA, USA). The signal corresponding to nonspecific binding was previously subtracted from the basal and agonist-stimulated binding. Then, the data was expressed as the percentage of stimulation over basal following the formula: ([^35^S]­GTPγS agonist-stimulated binding) × 100/([^35^S]­GTPγS basal binding)-100.

### Molecular Docking Simulations

Molecular docking calculations were performed using GOLD (CCDC Discovery 2020) and the ChemScore fitness function.^52,53^ The structure of human cannabinoid receptor CB_1_ was taken from PDB 5TGZ.^43^ The receptor was prepared for docking using UCSF Chimera to add hydrogen atoms.^54^ The docking cavity was centered on the α-carbon of Ser383 and allowed to extend in a spherical surrounding region with a 15 Å radius. Ligand coordinates were optimized with Gaussian 16 using the ωB97X-D functional^55^ and 6-31G­(d) basis set. The number of genetic algorithm runs was set to 20. Flexible ligand docking was performed, allowing ligand torsions around rotatable bonds and keeping receptor coordinates frozen to crystallographic values.

### Molecular Dynamics Simulations

Amber 22 was used to run all MD simulations using force field ff19SB (receptor),^56^ GAFF2 (ligand),^57^ and OPC (water).^58^ The ligand–receptor complex obtained with molecular docking was neutralized by incorporating explicit Cl^–^ counterions and enclosed in a cubic water box, surrounded by a 10 Å buffer of molecules. The region comprised between residues Val306 and Pro332, which is not resolved in the crystallographic structure of CB_1_, was not modeled, and a chain break was introduced. A geometry optimization approach in two stages was implemented. The first stage minimizes only the positions of solvent molecules and ions, and the second stage is an unrestrained minimization of all atoms in the simulation cell. Subsequently, the system was heated by incrementing the temperature from 0 to 300 K under a constant pressure of 1 atm and periodic boundary conditions. To control and equalize the temperature, we used the Andersen temperature coupling scheme,^59,60^ and Harmonic restraints of 10 kcal mol^–1^ Å^–2^ were applied to the solute. The time step was kept at 1 fs during the heating stages, allowing for the self-adjustment of potential inhomogeneities. For further equilibration and production, the SHAKE^61^ algorithm was employed, with a 2 fs time step. The modeling of long-range electrostatic effects was performed using the particle mesh Ewald method.^62^ A cutoff of 8 Å was applied to the Lennard-Jones interactions. The equilibration of the system was performed for 2 ns at constant volume and at a temperature of 300 K, and production was run as a 100 ns trajectory under the same conditions.

### Statistical Analysis

Data from radioligand affinity assays were analyzed by using nonlinear regression. A comparison of fits was performed for every curve and the preferred model (one site vs two sites) was chosen in each case. Curves were fitted to the pooled data from multiple experiments. For the evaluation of data from autoradiographic assays, the Kruskal–Wallis test followed by Dunn’s post hoc tests for multiple comparisons was performed. For statistical significance, the threshold was set at *p* = 0.05. The number of replicates or animal sample number used in each case is stated in the figure legends. GraphPad Prism 9 (GraphPad Software) was employed for data analysis and presentation.

## Supplementary Material



## Notes

Additional experimental details regarding the synthesis of the compounds; IC_50_ values of each compound; [^3^H]­CP55,940 binding values for each brain area analyzed in the different conditions; [^35^S]­GTPγS functional assays in membrane homogenates from rat cortical tissue and in CHO cells overexpressing the human CB_1_ receptor with UVI3502; [^35^S]­GTPγS binding values for each brain area analyzed in the different conditions; and docking poses of UVI3502 and related analogues on human CB_1_ receptor as well as the chemical structures of these compounds, together with their docking scores on human CB_1_ receptor (PDF)

## ¶

Institut de Neurociències, Universitat de Barcelona (NeuroUB), Diagonal Avenue 643, 08028, Barcelona, Spain

## ∇

Department of Translational Neuroscience, Barrow Neurological Institute, 2910 N third Avenue, 85013, Phoenix, AZ, USA.

## Abbreviations

Δ9-THC: Δ-9-tetrahydrocannabinol
[^35^S]­GTPγS: [^35^S]­guanosine 5′-*O*-3-thiotriphosphate
AD: Alzheimer’s disease
ADME: absorption, distribution, metabolism, and excretion
BSA: bovine serum albumin
CB_1_: cannabinoid receptor 1
CB_2_: cannabinoid receptor 2
CNS: central nervous system
DS: Down’s syndrome
DTT: DL-dithiothreitol
eCB: endocannabinoid
GDP: guanosine 5′-diphosphate
GPCR: G protein-coupled receptor
GTPγS: guanosine 5′-*O*-3-thiotriphosphate
HD: Huntington’s disease
LPA: lysophosphatidic acid
MCR: multicomponent reaction
NBM: nucleus basalis magnocellularis
PD: Parkinson’s disease
