# Supplementary material for: Synthesis and Pharmacological Characterization of a Novel Cannabinoid Receptor 1 Antagonist
Source: ACS Omega. 2025 Jun 2;10(22):22747–59. doi: 10.1021/acsomega.4c11355 (PMC12163822; doi:10.1021/acsomega.4c11355)
Supplement: Supplementary file 1 [file ao4c11355_si_001.pdf]

Synthesis and pharmacological characterization of a novel cannabinoid receptor 1 (CB_1) antagonist

Iker Bengoetxea de Tena[1], Gorka Pereira-Castelo[1], Jonatan Martínez-Gardeazabal[1], Marta Moreno-Rodríguez[1], Iván Manuel[1,2], Claudio Martínez[3], Belén Vaz[3], Javier González-Ricarte[3], Rosana Álvarez[3], Angel Torres-Mozas[4], Francesca Peccati[4,5], Gonzalo Jiménez-Osés[4,5], Ángel Rodríguez De Lera[3], Rafael Rodríguez-Puertas[1,2].*

[1]*Department of Pharmacology, Faculty of Medicine and Nursing, University of the Basque Country (UPV/EHU), Leioa University Campus, Sarriena s/n., 48940, Leioa, Spain*

[2]*Neurodegenerative Diseases, BioBizkaia Health Research Institute, Biobizkaia Building, Cruces Square, 48903, Barakaldo, Spain*

[3]*CINBIO and Department of Organic Chemistry, Faculty of Chemistry, University of Vigo, Experimental Sciencies Building, Lagoas-Marcosende University Campus, 36310, Vigo, Spain*

[4]*Basque Research and Technology Alliance (BRTA), Bizkaia Technology Park, Building 800, 48160, Derio, Spain*

[5]*Ikerbasque, Basque Foundation for Science, 48013, Bilbao, Spain*

SUPPORTING INFORMATION

General information for the synthesis of the novel compounds

THF was dried using a Puresolv solvent purification system. Commercial anhydrous 1,4-dioxane (99.8%) and DMF (≥99.8%) were kept over 4 Å MS. All other reagents were commercial compounds of the highest purity available. For reactions that require heating we used the Heat-On block system of Radleys. New compounds were fully characterized by their ^{1}H and ^{13}C NMR, IR, and HRMS spectral properties. Unless otherwise indicated, routine NMR spectra were obtained at 25 °C on a Bruker ARX-400 spectrometer (400.16 MHz for ^{1}H and 100.62 MHz for ^{13}C) using $CDCl_3$, CD_3OD, and DMSO-d_6 as solvents and internal reference ($CDCl_3$ δ 7.26 for ^{1}H and δ 77.0 for ^{13}C, CD_3OD δ 3.31 for ^{1}H and δ 49.0 for ^{13}C, DMSO-d_6 δ 2.50 for ^{1}H and δ 39.5 for ^{13}C). Chemical shifts (δ) are reported in ppm and coupling constants (*J*) are given in hertz (Hz). The proton spectra are reported as follows: multiplicity, coupling constant *J*, number of protons. The DEPT sequence was routinely used for ^{13}C multiplicity assignment. Multiplicity in the ^{13}C NMR spectral data refers to the attached hydrogens. Additionally, a combination of COSY, HSQC, and HMBC NMR experiments were used for structural assignments. Infrared spectra (IR) data were obtained from a thin film deposited onto a NaCl glass and were measured on a Jasco FT/IR 4100 in the interval between 4000 and 600 cm^{-1} with a 4 cm^{-1} resolution; data include only characteristic absorptions. Electrospray ionization (ESI) mass spectra were obtained on a micrOTOF focus mass spectrometer (Bruker Daltonics) using an ApolloII (ESI) source with a voltage of 4500 V applied to the capillary. Electron impact (EI) mass spectra were obtained on a Hewlett-Packard HP59970 instrument operating at 70 eV. For UPLC-QTOF, chromatographic separation was done with an Acquity UPLC BEH C18 1.7 µm, 50 mm ×

2.1 mm column, and H_2O/HCO_2H (99.9:0.1, *v/v*) or $MeOH/HCO_2H$ (99.9:0.1, *v/v*) as eluent mixture; the ionization source was electrospray in positive mode (ESI^+) with a voltage of 15 V; the range of masses in acquisition was 50–1200 u in SCAN mode. Flash-column chromatography was carried out in an automated system, using silica gel (230–400 mesh). Analytical thin layer chromatography (TLC) was performed on aluminum plates with Merck Kieselgel $60F_{254}$ and visualized by UV irradiation (254 nm) or by staining with a solution of phosphomolybdic acid, $KMnO_4$, or *p*-anisaldehyde. Melting points were measured in a Büchi B-540 apparatus in open capillary tubes.

Ethyl (2-Iodophenyl)carbamate (2a)[1].

Ethyl (2-Iodo-4-methylphenyl)carbamate (2b).

General procedure for aniline protection as carbamate. To a cooled (0 ºC) suspension of K_2CO_3 (0.36 g, 2.58 mmol) and 2-iodo-4-methylaniline **1b** (0.50 g, 2.15 mmol) in THF (11 mL), was added dropwise ethyl chloroformate (0.23 mL, 2.36 mmol) and the mixture was stirred at room temperature for 3 h. The reaction was poured over H_2O and the mixture was extracted with CH_2Cl_2 (3x). The combined organic layers were washed with a saturated aqueous solution of $NaHCO_3$ and dried with anhydrous Na_2SO_4. The solvent was evaporated, and the residue was adsorbed on silica gel before purification by flash-column chromatography (silica gel, gradient from 100:0 to 80:20 *v/v*, *n*-hexane/EtOAc) to afford 0.49 g (74% yield) of **2b**. **m.p.**: 52-54 °C (hexane/EtOAc). **^{1}H NMR** (400.13 MHz, $CDCl_3$): δ 7.90 (d, *J* = 8.4 Hz, 1H), 7.61 (dd, *J* = 2.0, 0.9 Hz, 1H), 7.16 (d, *J* = 8.4, 2.0 Hz, 1H), 6.84 (br s, 1H), 4.26 (q, *J* = 7.1 Hz, 2H), 2.29 (d, *J* = 0.7 Hz, 3H), 1.35 (t, *J* = 7.1 Hz, 3H) ppm. **^{13}C NMR** (100.61 MHz, $CDCl_3$): δ 153.6 (s), 139.0 (d), 136.0 (s), 135.0 (s), 129.3 (d), 120.3 (s), 89.1 (d), 61.5 (t), 20.2 (q), 14.5 (q) ppm. **IR** (NaCl): ν 3390 (w, N-H),

1736 (s, C=O), 1520 (s), 1220 (s, C-O) cm^{-1}. **HRMS** (ESI^+): Calcd for $C_{10}H_{13}INO_2$ ($[M+H]^+$), 305.9985; found, 305.9975.

Ethyl (2-Iodo-4-methoxyphenyl)carbamate (2c). Following the general procedure for aniline protection as carbamate described above, the reaction of K_2CO_3 (0.14 g, 1.04 mmol), 2-iodo-4-methoxyaniline **1c** (0.20 g, 0.80 mmol) and ethyl chloroformate (0.09 mL, 0.96 mmol) in THF (4 mL) afforded, after purification by flash-column chromatography (silica gel, gradient from 100:0 to 80:20 *v/v*, *n*-hexane/EtOAc), 0.21 g (81% yield) of **2c**. **m.p.**: 79-80 ºC (hexane/EtOAc). **^{1}H NMR** (400.13 MHz, $CDCl_3$): δ 7.81 (br d, J = 9.0 Hz, 1H), 7.30 (d, J = 2.9 Hz, 1H), 6.90 (dd, J = 9.0, 2.9 Hz, 1H), 6.65 (br s, 1H), 4.23 (q, J = 7.1 Hz, 2H), 3.76 (s, 3H), 1.32 (t, J = 7.1 Hz, 3H) ppm. **^{13}C NMR** (100.61 MHz, $CDCl_3$): δ 156.4 (s), 154.0 (s), 132.1 (s), 123.9 (d), 122.2 (d), 115.1 (d), 90.9 (s), 61.6 (t), 55.8 (q), 14.7 (q) ppm. **IR** (NaCl): ν 3279 (s, N-H), 2978 (w), 2938 (w), 2904 (w), 2838 (w), 1697 (s, C=O), 1528 (s), 1401 (w), 1283 (s, C-O-C), 1240 (s, C-O), 1216 (s, C-O), 1034 (w), 1024 (w), 854 (w) cm^{-1}. **HRMS** (ESI^+): Calcd for $C_{10}H_{13}INO_3$ ($[M+H]^+$), 321.9935; found, 321.9931.

Ethyl (4-Bromo-2-iodophenyl)carbamate (2d). Following the general procedure for aniline protection as carbamate described above, the reaction of 4-bromo-2-iodoaniline **1d** (0.50 g, 1.68 mmol), ethyl chloroformate (0.19 mL, 2.01 mmol) and K_2CO_3 (301.5 mg, 2.18 mmol) in THF (8.4 mL) afforded, after purification by flash-column chromatography (silica gel, gradient from 100:0 to 80:20 *v/v*, *n*-hexane/EtOAc), 0.55 g (88% yield) of **2d**. **m.p.**: 105-106 °C (*n*-hexane/EtOAc). **^{1}H NMR** (400.13 MHz, $CDCl_3$): δ 8.00 (d, J = 8.9 Hz, 1H), 7.90 (d, J = 2.3 Hz, 1H), 7.47 (dd, J = 8.9, 2.3 Hz, 1H), 6.93 (br s, 1H), 4.27 (q, J = 7.1 Hz, 2H), 1.36 (t, J = 7.1 Hz, 3H) ppm. **^{13}C NMR** (100.61 MHz, $CDCl_3$): δ 153.3 (s),

140.5 (d), 137.8 (s), 132.2 (d), 120.9 (d), 116.2 (s), 88.8 (s), 61.8 (t), 14.5 (q) ppm. **IR** (NaCl): ν 3294 (s, N-H), 2980 (w, C-H), 2956 (w, C-H), 2929 (w, C-H), 1690 (s, C=O), 1562 (s), 1518 (s), 1075 (s, C-Br) cm^{-1}. **HRMS** (ESI^+): Calcd for $C_9H_9{}^{79}BrINO_2$ ($[M+H]^+$), 368.8861; found, 368.8865; Calcd for $C_9H_9{}^{81}BrINO_2$ ($[M+H]^+$), 370.8841; found, 370.8845.

Ethyl (4-Methoxycarbonyl-2-iodophenyl)carbamate (2e)[2].

Ethyl (*E*)-4-(2-Ethynylphenylamino)-4-oxobut-2-enoate (4a).

General procedure for aniline protection as amide. To a suspension of 2-ethynylaniline **3a** (0.49 mL, 4.27 mmol) and $NaHCO_3$ (0.72 g, 8.54 mmol) in dioxane (8 mL) at 0 °C was added dropwise ethyl fumaroyl chloride (0.71 mL, 6.40 mmol). After 23 h at 25 ºC, the reaction was poured into water and the mixture was extracted with EtOAc (3x). The combined organic layers were washed with water and dried. The solvent was evaporated, and the residue was adsorbed on silica gel before purification by flash-column chromatography (silica gel, gradient from 100:0 to 80:20 *v/v*, *n*-hexane/EtOAc), to afford 0.94 g (90% yield) of **4a**. **m.p.**: 139 °C (Et_2O). **^{1}H NMR** (400.13 MHz, $CDCl_3$): δ 8.51 (d, J = 8.4 Hz, 1H), 8.19 (br s, 1H), 7.49 (dd, J = 7.8, 1.5 Hz, 1H), 7.40 (td, J = 8.0, 1.6 Hz, 1H), 7.10 (td, J = 7.6, 1.1 Hz, 1H), 7.07 (d, J = 15.3 Hz, 1H), 6.96 (d, J = 15.3 Hz, 1H), 4.29 (q, J = 7.1 Hz, 2H), 3.58 (s, 1H), 1.35 (t, J = 7.1 Hz, 3H) ppm. **^{13}C NMR** (100.61 MHz, $CDCl_3$): δ 165.4 (s), 161.5 (s), 139.0 (d), 136.4 (s), 132.4 (d), 131.9 (d), 130.4 (d), 124.3 (d), 119.7 (d), 111.2 (s), 85.3 (s), 79.0 (d), 61.5 (t), 14.2 (q) ppm. **IR** (NaCl): ν 3289 (w, N–H), 3247 (s, ≡C–H), 1712 (s, C=O), 1641 (s, C=O) cm^{-1}. **HRMS** (ESI^+): Calcd for $C_{14}H_{13}NO_3$ ($[M]^+$), 243.0895; found, 243.0893.

Ethyl (*E*)-4-(2-Ethynyl-4-methylphenylamino)-4-oxobut-2-enoate (4b). Following the general procedure for aniline protection as amide described above, the reaction of ethyl fumaroyl chloride (0.71 mL, 6.16 mmol), 2-ethynyl-4-methylaniline **3b** (0.34 mL, 4.11 mmol) and $NaHCO_3$ (690.4 mg, 8.22 mmol) in dioxane (14 mL) afforded, after purification by flash-column chromatography (silica gel, gradient from 100:0 to 80:20 *v/v*, *n*-hexane/EtOAc), 0.67 g (66% yield) of **4b**. **m.p.**: 138-140 °C (*n*-hexane/EtOAc). **^{1}H NMR** (400.13 MHz, $CDCl_3$): δ 8.38 (d, J = 8.5 Hz, 1H), 8.12 (br s, 1H), 7.30 (s, 1H), 7.20 (d, J = 8.0 Hz, 1H), 7.06 (d, J = 15.3 Hz, 1H), 6.94 (d, J = 15.3 Hz, 1H), 4.29 (q, J = 7.1 Hz, 2H), 3.53 (s, 1H), 2.30 (s, 3H), 1.34 (t, J = 7.1 Hz, 3H) ppm. **^{13}C NMR** (100.61 MHz, $CDCl_3$): δ 165.5 (s), 161.4 (s), 136.7 (s), 136.6 (d), 134.1 (s), 132.7 (d), 131.7 (d), 131.2 (d), 119.6 (d), 111.1 (s), 84.8 (s), 79.2 (d), 61.5 (t), 20.8 (q), 14.3 (q) ppm. **IR** (NaCl): ν 3280 (w, N–H), 3252 (s, C≡C-H), 2980 (w, C-H), 1711 (s, C=O), 1667 (w, C=O), 1642 (s, N-H), 1534 (w, N-H), 1298 (w, C-O-C), 675 (w) cm^{-1}. **HRMS** (ESI^+): Calcd. for $C_{15}H_{16}NO_3$ ($[M+H]^+$); 258.1125; found, 258.1132.

Ethyl (*E*)-4-(2-Ethynyl-4-fluorophenylamino)-4-oxobut-2-enoate (4c). Following the general procedure for aniline protection as amide described above, the reaction of ethyl fumaroyl chloride (0.39 mL, 3.40 mmol), 2-ethynyl-4-fluoroaniline **3c** (0.32 g, 2.28 mmol) and $NaHCO_3$ (0.38 g, 4.60 mmol) in dioxane (8 mL) afforded, after purification by flash-column chromatography (silica gel, gradient from 100:0 to 80:20 *v/v*, *n*-hexane/EtOAc), 0.43 g (72% yield) of a white solid identified as **4c**. **m.p.:** 170-172 °C (*n*-hexane/EtOAc). **^{1}H NMR** (400.13 MHz, $CDCl_3$): δ 8.50 (dd, J = 9.2 Hz, and $^4J_{H,F}$ = 5.2 Hz, 1H), 8.11 (br s, 1H), 7.21 (dd, J = 3.0 Hz, and $^3J_{H,F}$ = 8.4 Hz, 1H), 7.13 (ddd, J = 9.2, 3.0 Hz, and $^3J_{H,F}$ = 8.0 Hz, 1H), 7.08 (d, J = 15.3 Hz, 1H), 6.97 (d, J = 15.3 Hz, 1H), 4.31 (q, J = 7.1 Hz, 2H),

3.63 (s, 1H), 1.37 (t, J = 7.1 Hz, 3H) ppm. **^{13}C NMR** (100.61 MHz, $CDCl_3$): δ 165.3 (s), 161.3 (s), 158.5 (s, $^{1}J_{C,F}$ = 245.7 Hz), 136.1 (d), 135.4 (s, $^{4}J_{C,F}$ = 2.9 Hz), 132.0 (d), 121.5 (d, $^{3}J_{C,F}$ = 8.3 Hz), 118.7 (d, $^{2}J_{C,F}$ = 18.7 Hz), 117.5 (d, $^{2}J_{C,F}$ = 22.1 Hz), 112.8 (s, $^{3}J_{C,F}$ = 9.2 Hz), 85.9 (s), 77.9 (d, $^{5}J_{C,F}$ = 2.9 Hz), 61.5 (t), 14.1 (q) ppm. **IR** (NaCl): ν 3282 (w, N-H), 3245 (s, N-H), 3077 (w), 1711 (s, C=O), 1668 (w, C=O), 1644 (s), 1538 (w), 1302 (w, C-O-C), 674 (w) cm^{-1}. **HRMS** (ESI^{+}): Calcd. for $C_{14}H_{13}FNO_3$ ([M+H]$^{+}$), 262.0874; found, 262.0871.

Ethyl (*Z*)-7-(2-Ethoxy-2-oxoethylidene)-dihydrobenzo[2,3]azepino[4,5-*b*]indole-12(5*H*)-carboxylate (5a, UVI3508)

General procedure for one-pot Sonogashira-Heterocyclization-oxidative Heck reaction. To a degassed solution of ethyl (2-iodophenyl)carbamate **2a** (0.20 g, 0.69 mmol) and alkyne **4a** (0.33 g, 1.37 mmol) in DMF (7.6 mL) were sequentially added Et_3N (0.43 mL, 3.09 mmol), $PdCl_2(PPh_3)$ (0.048 g, 0.069 mmol), CuI (0.052 g, 0.28 mmol) and PPh_3 (0.018 g, 0.069 mmol). The mixture was stirred at 60 °C for 12 h. Then, the flask was opened to air and the reaction mixture was stirred at 100 °C for 15 h. A water/brine solution (90:10 *v/v*) was added and the mixture was extracted with EtOAc (3x). The combined organic layers were dried. The solvent was evaporated, and the residue was adsorbed on silica gel before purification by column chromatography (silica gel, gradient from 100:0 to 60:40 *v/v*, hexane/EtOAc) to afford 0.15 g (52% yield) of **5a**.[6] **^{1}H NMR** (400.13 MHz, DMSO-d_6, 333 K): δ 8.83 (s, 1H), 8.20 (d, J = 8.3 Hz, 1H), 7.79 (dd, J = 7.7, 1.2 Hz, 1H), 7.45 (ddd, J = 8.5, 7.3, 1.3 Hz, 1H), 7.41 – 7.31 (m, 3H), 7.28 (d, J = 7.9 Hz, 1H), 7.20 (td, J = 7.5, 1.4 Hz, 1H), 6.31 (s, 1H), 4.35 (q, J = 7.2 Hz, 2H), 4.25 (q, J = 7.1 Hz, 2H), 1.31 (t, J = 7.1 Hz, 3H), 1.21 (t, J = 7.1 Hz, 3H) ppm. **^{13}C NMR** (100.61 MHz, DMSO-d_6, 333 K):

δ 168.9 (s), 164.9 (s), 151.7 (s), 139.2 (s), 138.8 (s), 134.0 (s), 133.1 (s), 130.1 (d), 128.9 (d), 126.6 (d), 126.6 (s), 125.2 (d), 124.3 (d), 124.1 (d), 124.1 (s), 123.0 (d), 121.9 (s), 119.3 (d), 115.6 (d), 63.9 (t), 61.3 (t), 14.1 (q), 14.0 (q) ppm.

Ethyl (*Z*)-7-(2-Ethoxy-2-oxoethylidene)-3-methyl-6-oxo-6,7-dihydrobenzo[2,3]azepino[4,5-*b*]indole-12(5*H*)-carboxylate (5b, UVI3511). Following the general procedure for the one-pot Sonogashira-heterocyclization- oxidative Heck reaction described above, the reaction of iodide **2a** (0.20 g, 0.69 mmol), alkyne **4b** (0.35 g, 1.37 mmol), Et_3N (0.43 mL, 3.09 mmol), $PdCl_2(PPh_3)_2$ (0.048 g, 0.069 mmol), CuI (0.052 g, 0.28 mmol) and PPh_3 (0.018 g, 0.069 mmol) in DMF (7.6 mL) afforded, after purification by flash-column chromatography (silica gel, gradient from 100:0 to 60:40 *v/v*, *n*-hexane/EtOAc), 0.22 g (75% yield) of **5b**. **m.p.:** 255-257 °C (*n*-hexane/EtOAc). **^{1}H NMR** (400.13 MHz, $CDCl_3$, 323 K): δ 8.67 (s, 1H), 8.20 (d, *J* = 8.3 Hz, 1H), 7.78 (dt, *J* = 7.8, 1.1 Hz, 1H), 7.43 (ddd, *J* = 8.4, 7.2, 1.3 Hz, 1H), 7.35 (t, *J* = 7.5 Hz, 1H), 7.19 – 7.12 (m, 2H), 7.15 (s, 1H), 6.28 (s, 1H), 4.35 (q, *J* = 7.1 Hz, 2H), 4.25 (q, *J* = 7.1 Hz, 2H), 2.36 (s, 3H), 1.30 (t, *J* = 7.1 Hz, 3H), 1.21 (t, *J* = 7.1 Hz, 3H) ppm. **^{13}C NMR** (100.61 MHz, $CDCl_3$, 323 K): δ 168.9 (s), 164.9 (s), 151.7 (s), 139.4 (s), 137.1 (s), 134.2 (s), 133.6 (s), 130.9 (s), 130.2 (d), 129.8 (d), 126.7 (s), 126.5 (d), 125.2 (d), 124.2 (d), 124.1 (s), 122.9 (d), 121.9 (s), 119.3 (d), 115.5 (d), 63.8 (t), 61.2 (t), 21.4 (q), 14.1 (q), 14.0 (q) ppm. **IR** (NaCl): v 3196 (w, N-H), 2981(s, C-H), 2930 (w, C-H), 1731 (s, C=O), 1666 (s, C=O), 1585 (w, N-C=O), 1505 (s), 1454 (w, $-CH_3$), 1371 (s), 1307 (s), 1226 (s, C-O-C), 759 (s) cm^{-1}. **HRMS** (ESI$^+$): Calcd. for $C_{24}H_{23}N_2O_5$ ($[M+H]^+$), 419.1601; found, 419.1588.

Ethyl (*Z*)-7-(2-Ethoxy-2-oxoethylidene)-3-methyl-9-bromo-6-oxo-6,7-dihydrobenzo[2,3]azepino[4,5-*b*]indole-12(5*H*)-carboxylate (5c, UVI3507). Following

the general procedure for the one-pot Sonogashira-heterocyclization-oxidative Heck reaction described above, the reaction of iodide **2d** (0.15 g, 0.41 mmol), alkyne **4b** (0.21 g, 0.81 mmol), Et_3N (0.25 mL, 1.82 mmol), $PdCl_2(PPh_3)_2$ (29 mg, 0.041 mmol, CuI (31 mg, 0.031 mmol) and PPh_3 (11 mg, 0.041 mmol) in DMF (4.5 mL) afforded, after purification by flash-column chromatography (silica gel, gradient from 100:0 to 60:40 *v/v*, *n*-hexane/EtOAc), 0.11 g (55% yield) of **5c**. **m.p.:** 271-274 °C (*n*-hexane/EtOAc). ^{1}**H NMR** (400.13 MHz, $CDCl_3$, 298 K): δ 8.66 (br s, 1H), 8.10 (d, *J* = 8.9 Hz, 1H), 7.88 (d, *J* = 2.0 Hz, 1H), 7.52 (dd, *J* = 8.9, 2.0 Hz, 1H), 7.18 – 7.16 (m, 2H), 7.13 (s, 1H), 6.27 (s, 1H), 4.40 – 4.29 (m, 2H), 4.25 (q, *J* = 7.1 Hz, 2H), 2.35 (s, 3H), 1.31 (t, *J* = 7.1 Hz, 3H), 1.20 (t, *J* = 7.1 Hz, 3H) ppm. ^{13}**C NMR** (100.61 MHz, $CDCl_3$, 298 K): δ 168.6 (s), 164.7 (s), 151.3 (s), 138.9 (s), 137.4 (s), 135.2 (s), 133.8 (s), 130.9 (s), 130.3 (d), 130.2 (d), 129.3 (d), 128.1 (s), 125.4 (d), 123.5 (s), 123.1 (d), 121.9 (d), 120.6 (s), 117.6 (s), 117.0 (d), 64.0 (t), 61.2 (t), 20.9 (q), 14.0 (q), 13.8 (q) ppm. **IR** (NaCl): ν 2980 (s, C-H), 2932 (w, C-H), 1731 (s, C=O), 1666 (s, C=O), 1595 (w, N-C=O), 1505 (s), 1449 (w), 1372 (s), 1262 (s), 1227 (s), 1176 (s), 1043 (s) cm^{-1}. **HRMS** (ESI^+): Calcd. for $C_{24}H_{22}{}^{79}BrN_2O_5$ ($[M+H]^+$), 497.0707; found, 497.0691.

Ethyl (*Z*)-7-(2-Ethoxy-2-oxoethylidene)-2-fluoro-9-methyl-6-oxo-6,7-dihydrobenzo[2,3]azepino[4.5-b]indole-12(5*H*)-carboxylate (5d, UVI3503). Following the general procedure for the one-pot Sonogashira-heterocyclization-oxidative Heck reaction described above, the reaction of iodide **2b** (0.15 g, 0.49 mmol), alkyne **4c** (0.26 g, 0.98 mmol), Et_3N (0.31 mL, 2.21 mmol), $PdCl_2(PPh_3)_2$ (34 mg, 0.049 mmol), CuI (38 mg, 0.20 mmol) and PPh_3 (13 mg, 0.049 mmol) in DMF (5.5 mL) afforded, after purification by flash-column chromatography (silica gel, gradient from 100:0 to 60:40 *v/v*, *n*-

hexane/EtOAc), 0.1 g (47% yield) of **5d**. **m.p.:** 226-227 °C (MeOH). **^{1}H NMR** (400.13 MHz, $CDCl_3$, 323 K): δ 9.27 (s, 1H), 8.08 (d, *J* = 8.5 Hz, 1H), 7.56 (s, 1H), 7.33 – 7.27 (m, 2H), 7.08 – 6.99 (m, 2H), 6.29 (s, 1H), 4.38 (q, *J* = 7.1 Hz, 2H), 4.24 (q, *J* = 7.1 Hz, 2H), 2.48 (s, 3H), 1.30 (t, *J* = 7.1 Hz, 3H), 1.26 (t, *J* = 7.1 Hz, 3H) ppm. **^{13}C NMR** (100.61 MHz, $CDCl_3$, 323 K): δ 169.2 (s), 164.8 (s), 158.9 (s, $^1J_{C,F}$ = 244.1 Hz), 151.4 (s), 139.3 (s), 137.2 (s), 134.2 (s), 133.0 (s, $^4J_{C,F}$ = 1.5 Hz), 129.6 (s, $^4J_{C,F}$ = 2.7 Hz), 128.4 (d), 126.7 (s), 126.0 (s, $^3J_{C,F}$ = 9.1 Hz), 126.3 (d), 124.9 (d, $^3J_{C,F}$ = 8.5 Hz), 122.7 (s), 119.2 (d), 116.2 (d, $^2J_{C,F}$ = 20.6 Hz), 115.9 (d, $^2J_{C,F}$ = 19.0 Hz), 115.5 (d), 63.9 (t), 61.3 (t), 21.4 (q), 14.1 (q), 14.0 (q) ppm. **IR** (NaCl): ν 3199 (w, N-H), 3074 (w, N-H), 2980 (w, C-H), 2938 (w, C-H), 1727 (s, C=O), 1666 (s, C=O), 1502 (m), 1396 (m), 1307 (m), 1185 (s), 1041 (m) cm^{-1}. **HRMS** (ESI$^+$): Calcd. for $C_{24}H_{22}FN_2O_5$ ([M+H]$^+$); 437.1507; found, 437.1508.

12-Ethyl 9-Methyl (*Z*)-7-(2-Ethoxy-2-oxoethylidene)-2-methyl-6-oxo-6,7-dihydrobenzo[2,3]azepino[4,5-b]indole-12, 9(5*H*)-dicarboxylate (5e, UVI3501). Following the general procedure for the one-pot Sonogashira-heterocyclization-oxidative Heck reaction described above, the reaction of iodide **2e** (0.15 g, 0.43 mmol), alkyne **x.x** (0.22 g, 0.86 mmol), Et_3N (0.27 mL, 1.94 mmol), $PdCl_2(PPh_3)_2$ (30 mg, 0.043 mmol), CuI (33 mg, 0.17 mmol) and PPh_3 (11 mg, 0.043 mmol) in DMF (4.8 mL) afforded, after purification by flash-column chromatography (silica gel, gradient from 100:0 to 60:40 *v/v*, *n*-hexane/EtOAc), 0.11 g (51% yield) of **5e**. **m.p.:** 263-264 °C ($CHCl_3$). **^{1}H NMR** (400.13 MHz, $CDCl_3$, 323 K): δ 8.66 (s, 1H), 8.47 (d, *J* = 1.7 Hz, 1H), 8.23 (d, *J* = 8.8 Hz, 1H), 8.13 (dd, *J* = 8.8, 1.7 Hz, 1H), 7.19 – 7.17 (m, 2H), 6.30 (s, 1H), 4.36 (q, *J* = 7.1 Hz, 2H), 4.26 (q, *J* = 7.1 Hz, 2H), 3.97 (s, 3H), 2.36 (s, 3H), 1.32 (t, *J* = 7.1 Hz, 3H), 1.21 (t, *J* = 7.1 Hz, 3H) ppm. **^{13}C NMR** (100.61 MHz, $CDCl_3$, 323 K): δ 168.8 (s), 167.0 (s), 164.7 (s),

151.3 (s), 141.4 (s), 139.0 (s), 135.4 (s), 133.8 (s), 131.2 (s), 130.2 (d), 130.1 (d), 127.7 (d), 126.6 (s), 126.5 (s), 125.7 (d), 123.6 (s), 123.1 (d), 121.7 (s), 121.4 (d), 115.2 (d), 64.2 (t), 61.3 (t), 52.3 (q), 21.0 (q), 14.1 (q), 13.9 (q) ppm. **IR** (NaCl): ν 3278 (w, N-H), 3031 (w, C-H), 2995 (w, C-H), 1721 (s, C=O), 1665 (s, C=O), 1373 (m), 1263 (s), 1226 (s), 1079 (m) cm^{-1}. **HRMS** (ESI^+): Calcd. for $C_{26}H_{25}N_2O_7$ ($[M+H]^+$); 477.1656; found, 477.1656.

Ethyl (*Z*)-7-(2-Ethoxy-2-oxoethylidene)-9-methoxy-2-methyl-6-oxo-6,7-dihydrobenzo[2,3]azepino[4,5-*b*]indole-12(*5H*)-carboxylate (5f, UVI3502). Following the general procedure for the one-pot Sonogashira-heterocyclization-oxidative Heck reaction described above, the reaction of iodide **x.x** (0.15 g, 0.47 mmol), alkyne **x.x** (0.24 g, 0.93 mmol), Et_3N (0.29 mL, 2.10 mmol), $PdCl_2(PPh_3)_2$ (33 mg, 0.047 mmol), CuI (36 mg, 0.19 mmol) and PPh_3 (12 mg, 0.047 mmol) in DMF (5.2 mL) afforded, after purification by flash-column chromatography (silica gel, gradient from 100:0 to 60:40 *v/v*, *n*-hexane/EtOAc), 0.12 g (55% yield) of **5f**. **m.p.:** 159-160 °C (MeOH). **^{1}H NMR** (400.13 MHz, $CDCl_3$, 323 K): δ 9.35 (s, 1H), 8.08 (d, *J* = 9.1 Hz, 1H), 7.24 – 7.18 (m, 2H), 7.16 – 7.08 (m, 2H), 7.03 (dd, *J* = 9.1, 2.5 Hz, 1H), 6.25 (s, 1H), 4.32 (q, *J* = 7.1 Hz, 2H), 4.23 (q, *J* = 7.1 Hz, 2H), 3.88 (s, 3H), 2.34 (s, 3H), 1.28 (t, *J* = 7.1 Hz, 3H), 1.18 (t, *J* = 7.1 Hz, 3H) ppm. **^{13}C NMR** (100.61 MHz, $CDCl_3$, 323 K): δ 169.2 (s), 164.8 (s), 157.2 (s), 151.6 (s), 139.8 (s), 134.8 (s), 133.5 (s), 133.4 (s), 131.0 (s), 130.2 (d), 129.7 (d), 127.4 (s), 124.6 (d), 123.9 (s), 123.0 (d), 121.6 (s), 116.4 (d), 115.4 (d), 101.7 (d), 63.6 (t), 61.1 (t), 55.9 (q), 20.9 (q), 14.0 (q), 13.9 (q) ppm. **IR** (NaCl): ν 3289 (w, N-H), 3194 (w, N-H), 2980 (w, C-H), 2938 (w, C-H), 2835 (w, C-H), 1731 (s, C=O), 1664 (s, C=O), 1506 (m), 1480 (m), 1373 (m), 1252 (m), 1163 (m), 1031 (m), 772 (s) cm^{-1}. **HRMS** (ESI^+): Calcd. for $C_{25}H_{25}N_2O_6$ ($[M+H]^+$); 449.1707; found, 449.1701.

Ethyl 2-(6-Oxo-5,6,7,12-tetrahydrobenzo[2,3]azepino[4,5-*b*]indol-7-yl)acetate (7a, UVI3505). To a solution of benzoazepinone **5a** (0.23 g, 0.57 mmol) in EtOAc (38 mL) was added $Pd(OH)_2$ (0.42 g, 0.60 mmol) and the resulting mixture was stirred at 25 °C under H_2 atmosphere (previous cycles of vacuum/H_2 were performed) for 24 h. After completion, the reaction mixture was filtered through a pad of Celite® and the product was eluted with 80:20 *v/v* CH_2Cl_2/MeOH. The product thus obtained was used in the next step without further purification. The solid obtained was introduced in a Schlenk tube and dissolved in THF (6.4 mL). A solution of TBAF (1M in THF, 2.87 mL, 2.87 mmol) was added and the mixture was stirred at 80 °C for 21 h. Then, an aqueous saturated solution of NH_4Cl was added and the mixture was extracted with EtOAc (3x). The combined organic layers were dried over Na_2SO_4, the solvent was evaporated, and the residue was adsorbed on silica gel before purification by flash-column chromatography (silica gel, gradient from 100:0 to 90:10 *v/v*, CH_2Cl_2/MeOH) to afford 0.14 g (73% yield, two steps) of a yellow solid identified as **7a**. **m.p.:** 257-259 °C (CH_2Cl_2/MeOH). **^{1}H NMR** (400.13 MHz, DMSO-d_6, 333 K): δ 10.08 (s, 1H), 7.79 (d, *J* = 8.0 Hz, 1H), 7.66 (d, *J* = 8.0 Hz, 1H), 7.44 (d, *J* = 8.0 Hz, 1H), 7.43 – 7.37 (m, 1H), 7.33 – 7.27 (m, 2H), 7.17 (dd, *J* = 8.2, 7.0 Hz, 1H), 7.07 (t, *J* = 7.5 Hz, 1H), 4.05 – 3.92 (m, 3H), 3.06 (dd, *J* = 15.6, 8.5 Hz, 1H), 2.73 (dd, *J* = 15.6, 7.2 Hz, 1H), 1.10 (t, *J* = 7.1 Hz, 3H) ppm. **^{13}C NMR** (100.63 MHz, DMSO-d_6, 333 K): δ 170.8 (s), 170.4 (s), 137.3 (s), 134.9 (s), 132.1 (s), 127.9 (d), 126.6 (d), 125.8 (s), 123.3 (d), 122.3 (s), 121.7 (d), 121.5 (d), 118.9 (d), 118.2 (d), 111.3 (d), 109.7 (s), 59.6 (t), 39.5 (d), 33.0 (t), 13.6 (q) ppm. **IR** (NaCl): ν 3301 (w, N-H), 3048 (w), 2970 (w), 1722 (s, C=O), 1645 (s, NHC=O), 1183 (s, C-O-C) cm^{-1}. **HRMS** (ESI^+): Calcd. for $C_{20}H_{19}N_2O_3$ ($[M+H]^+$); 335.1390; found, 335.1392.

Ethyl 2-(6-Methyl-9,14-dihydrobenzo[2,3][1,2,4]triazolo[4',3':1,7]azepino[4,5-*b*]indol-9-yl)acetate (9, UVI8113). Into a round-bottomed flask was added benzoazepinone **7a** (0.13 g, 0.39 mmol), Lawesson's reagent (0.32 g, 0.79 mmol) and THF (5.8 mL). The mixture was stirred at 60 °C for 3 h. The mixture was then diluted with CH_2Cl_2 and a saturated aqueous solution of $NaHCO_3$ was added. After extraction with CH_2Cl_2 (3x), the combined organic layers were washed with water (1x) and brine (1x). The solution was then dried over anhydrous Na_2SO_4, filtered, and concentrated. The residue was adsorbed on silica gel before purification by flash-column chromatography (silica gel, gradient from 100:0 to 95:5 *v/v*, CH_2Cl_2/MeOH) to afford 0.13 g (94%) of the thioacetamide, which was directly used in the next transformation.

Hydrazine hydrate was added dropwise to a stirred solution of the thioacetamide (22 mg, 0.062 mmol) in THF (0.3 mL) at 20 °C. After 2 h, the solution was concentrated under reduced pressure and the residue was treated with triethyl orthoacetate (0.40 mL, 2.17 mmol). The mixture was heated at 80 °C for 1 h. After cooling down to room temperature, a saturated aqueous solution of NH_4Cl and EtOAc were added, and the mixture was extracted with EtOAc (3x). The combined organic layers were dried over Na_2SO_4 and concentrated under reduced pressure. The residue was dissolved in the minimum amount of CH_2Cl_2 and Et_2O was added to form a precipitate. The solid was washed with Et_2O and dried to afford 20.2 mg (87% yield) of **9**. **^{1}H NMR** (400.13 MHz, Methanol-d_4, 333 K): δ 7.91 (dd, J = 7.8, 1.6 Hz, 1H), 7.74 (d, J = 8.4 Hz, 1H), 7.71 (dd, J = 8.1, 1.1 Hz, 2H), 7.65 (td, J = 7.6, 1.5 Hz, 1H), 7.59 (td, J = 7.7, 1.6 Hz, 1H), 7.43 (dt, J = 8.1, 0.9 Hz, 1H), 7.20 (t, J = 7.5 Hz, 1H), 7.12 (t, J = 7.6 Hz, 1H), 5.33 (t, J = 8.2 Hz, 1H), 3.90 (q, J = 6.5 Hz, 2H), 2.56 (dd, J = 14.7, 7.1 Hz, 1H), 2.49 (s, 3H), 2.42 (dd, J = 14.7, 8.5 Hz, 1H), 1.02 (t, J

= 7.3 Hz, 3H) ppm. **^{13}C NMR** (100.61 MHz, Methanol-*d*$_4$, 333 K): δ 171.8 (s), 159.1 (s), 154.2 (s), 138.8 (s), 131.5 (s), 130.1 (d), 129.5 (d), 129.1 (d), 128.0 (s), 127.6 (s), 127.2 (s), 126.7 (d), 124.4 (d), 121.0 (d), 119.3 (d), 116.5 (s), 112.6 (d), 61.9 (t), 37.3 (t), 31.9 (d), 14.2 (q), 12.3 (q) ppm. **IR** (NaCl): ν 3180 (w), 3108 (w), 3065 (w), 3023 (w), 2981 (w), 1733 (s, C=O), 1532 (m), 1496 (m), 1457 (m), 1414 (m), 1326 (m), 1264 (m), 1181 (m), 1031 (m) cm^{-1}. **HRMS** (ESI$^+$): Calcd. for $C_{22}H_{21}N_4O_2$ ([M+H]$^+$); 373.1659; found, 373.1665.

(*Z*)-2-(6-Oxo-5,12-dihydrobenzo[2,3]azepino[4,5-b]indol-7(6*H*)-ylidene)acetic Acid (6a, UVI3506). To a solution of **5a** (32 mg, 0.096 mmol) in THF (1.2 ml), a solution of LiOH H_2O (80.3 mg, 1.91 mmol) in H_2O (1.2 mL) was added and the mixture was stirred at 50 °C for 12 h. The reaction mixture was slowly quenched with $KHSO_4$ (1.0 M in water) to reach pH 4. Then, the mixture was extracted with EtOAc (3x) and the combined organic layers were washed with brine. The crude product was purified by flash-column chromatography (silica gel, gradient from 100:0 to 90:10 *v/v*, CH_2Cl_2/MeOH) to afford 25 mg (86% yield) of **6a**. **m.p.:** 258 – 260 °C (MeOH). **^{1}H NMR** (400.13 MHz, DMSO-*d*$_6$): δ 12.62 (s, 1H), 12.33 (s, 1H), 10.66 (s, 1H), 7.87 (dd, *J* = 7.9, 1.5 Hz, 1H), 7.72 (d, *J* = 8.0 Hz, 1H), 7.54 (d, *J* = 8.1 Hz, 1H), 7.40 (ddd, *J* = 8.5, 7.1, 1.5 Hz, 1H), 7.34 – 7.22 (m, 3H), 7.16 (ddd, *J* = 8.0, 7.0, 1.1 Hz, 1H), 6.19 (s, 1H) ppm. **^{13}C NMR** (100.61 MHz, DMSO-*d*$_6$): δ 166.9 (s), 166.4 (s), 138.6 (s), 137.9 (s), 134.3 (s), 133.5 (s), 128.7 (d), 127.3 (d), 125.2 (s), 123.9 (d), 123.2 (d), 122.9 (d), 122.1 (d), 121.5 (s), 120.4 (d), 118.3 (d), 112.0 (d), 110.6 (s) ppm. **IR** (NaCl): ν 3444 (s, O-H, N-H), 2997(s), 3913 (s), 1661 (s, C=O), 1437 (m), 1407(m) cm^{-1}. **HRMS** (ESI$^+$): Calcd. for $C_{18}H_{13}N_2O_3$ ([M+H]$^+$); 305.0920; found, 305.0921.

2-(6-Oxo-5,6,7,12-tetrahydrobenzo[2,3]azepino[4,5-b]indol-7-yl)acetic Acid (8, UVI3504). Following the general reaction for ester hydrolysis described above, the reaction of **5a** (14 mg, 0.040 mmol), LiOH H_2O (34 mg, 0.81 mmol) in THF (0.5 mL) and H_2O (0.5 mL) afforded, after purification by flash-column chromatography (silica gel, gradient from 100:0 to 90:10 *v/v*, CH_2Cl_2/MeOH), 10 mg (79% yield) of **8**. **^{1}H NMR** (400.13 MHz, DMSO-*d*$_6$): δ 10.55 (s, 1H), 7.79 (dd, *J* = 7.8, 1.5 Hz, 1H), 7.65 – 7.59 (m, 2H), 7.49 -7.40 (m, 1H), 7.29 (td, *J* = 7.6, 1.3 Hz, 1H), 7.26 – 7.20 (m, 2H), 7.11 (td, *J* = 7.5, 0.9 Hz, 1H), 6.88 (s, 1H), 5.68 (dd, *J* = 10.1, 4.9 Hz, 1H), 2.86 (dd. *J* = 16.0, 4.9 Hz, 1H), 2.43 (dd, *J* = 10.1, 6.0 Hz, 1H) ppm. **^{13}C NMR** (100.61 MHz, DMSO-*d*$_6$): δ 170.2 (s), 167.8 (s), 136.6 (s), 135.5 (s), 134.0 (s), 129.5 (s), 129.4 (d), 129.1 (d), 127.7 (s), 124.9 (d), 123.1 (s), 121.9 (d), 121.4 (d), 120.3 (d), 109.9 (d), 101.7 (d), 57.4 (d), 34.7 (t) ppm. **IR** (NaCl): v 3600-3000 (s, OH + NH), 3000.7 (m), 2916 (m), 1661 (s, C=O), 1437 (s), 1407 (s), 1314 (w) cm^{-1}. **HRMS** (ESI$^+$): Calcd. for $C_{18}H_{15}N_2O_3$ ([M+H]$^+$); 307.1077; found, 307.1078.

(*Z*)-2-(9-Methoxy-2-methyl-6-oxo-5,12-dihydrobenzo[2,3]azepino[4,5-b]indol-7(6*H*)-ylidene)acetic Acid (6f, UVI3509). To a solution of **5f** (40 mg, 0.089 mmol) in THF (1 mL), TBAF (0.45 mL, 0.45 mmol) was added. The resulting mixture was stirred at 80 °C for 21 h. Then, a saturated aqueous solution of NH_4Cl was added and extracted with EtOAc (3x). The combined organic layers were dried over anhydrous Na_2SO_4 and concentrated. The residue was purified by flash-column chromatography (silica gel, gradient from 100:0 to 90:10 *v/v*, CH_2Cl_2/MeOH) to afford 22 mg (71% yield) of **6f**. **m.p.:** 293 – 295 °C (MeOH). **^{1}H NMR** (400.13 MHz, DMSO-*d*$_6$): δ 12.65 (s, 1H), 8.21 (s, 1H), 7.66 (d, *J* = 8.1 Hz, 1H), 7.55 (d, *J* = 8.7 Hz, 1H), 7.50 (dd, *J* = 8.3, 1.8 Hz, 1H), 7.45 (d, *J* = 2.3 Hz, 1H), 7.10 (dd, *J* = 8.7, 2.3 Hz, 1H) 6.97 (s, 1H), 3.91 (s, 3H), 2.52 (s, 3H) ppm. **^{13}C NMR**

(100.61 MHz, DMSO-d_6): δ 168.6 (s), 155.7 (s), 154.8 (s), 140.4 (s), 139.1 (s), 139.0 (s), 136.6 (s), 134.4 (d), 133.1 (s), 131.8 (d), 126.2 (d), 125.7 (s), 123.4 (s), 116.3 (d), 113.2 (d), 108.5 (s), 103.9 (d), 103.2 (d), 55.9 (q), 21.1 (q) ppm. **IR** (NaCl): ν 3600-3000 (s, OH + N-H), 2999 (m), 2915 (m), 1662 (s, C=O), 1437 (w), 1407 (w) cm^{-1}. **HRMS** (ESI^+): Calcd. for $C_{20}H_{17}N_2O_4$ ($[M+H]^+$); 349.1183; found, 349.1184.

Table S1. IC_{50} and R^2 values from the competition curves performed with the 11 compounds using [^{3}H]CP55,940 *vs.* increasing concentrations ranging from 10^{-12} M to 10^{-4} M of each compound in membrane homogenates from rat brain cortex. Each IC_{50} value represents the mean of n= 2-5 independent experiments.

**Compound**	**IC_{50} (nM)**	**R^2**
UVI3501	0.0005 ± >10000	0.21
UVI3502	0.026 ± 0.43<br>772 ± 49.40	0.81
UVI3503	>10000	0.14
UVI3504	5908 ± 7.18	0.31
UVI3505	8.85 ± 14.62	0.23
UVI3506	7041 ± 17.98	0.18
UVI3507	0.04 ± 21.03	0.2
UVI3508	3.1e5 ± 101.65	0.7
UVI3509	>10000	0.17
UVI3511	0.03 ± 19.23	0.45
UVI8113	10.62 ± 43.35	0.13

Table S2. [^{3}H]CP55,940 binding in different brain areas of rats (n = 5) and mice (n = 5) showing the total binding, [^{3}H]CP55,940 binding in the presence of UVI3502 and [^{3}H]CP55,940 binding in the presence of SR141716A.

	[^{3}H]CP55,940 binding (fmol/mg t.e.)					
	Sprague-Dawley rats			Swiss mice		
**Brain region**	**Total**	**UVI3502**	**SR141716A**	**Total**	**UVI3502**	**SR141716A**
Amygdala	158 ± 28	136 ± 52	79 ± 18##	168 ± 41	154 ± 8	62 ± 37##
Cerebellum (gray matter)	724 ± 111	421 ± 126*	72 ± 23##	409 ± 115	409 ± 207	118 ± 22##
Cortex	133 ± 28	122 ± 25	66 ± 14#	81 ± 3	118 ± 18	65 ± 26
Hippocampus dorsal	299 ± 25	184 ± 26*	62 ± 10##	168 ± 15	181 ± 9	69 ± 29#
Globus pallidus	1632 ± 65	572 ± 40*	77 ± 15#	1074 ± 262	698 ± 16**	75 ± 31##
Nucleus basalis magnocellularis	180 ± 19	147 ± 18	80 ± 11##	237 ± 20	202 ± 19*	82 ± 31##
Striatum	560 ± 22	291 ± 18*	79 ± 25##	133 ± 15	159 ± 11	73 ± 13##
Substantia nigra	2720 ± 540	1364 ± 581*	70 ± 14##	1684 ± 387	1075 ± 22*	106 ± 118##

Total *vs*. UVI3502 (*), Total *vs*. SR141716A (#). Kruskal–Wallis test, *post-hoc* test Dunn's multiple comparison, *#$p < 0.05$; **##$p < 0.01$.

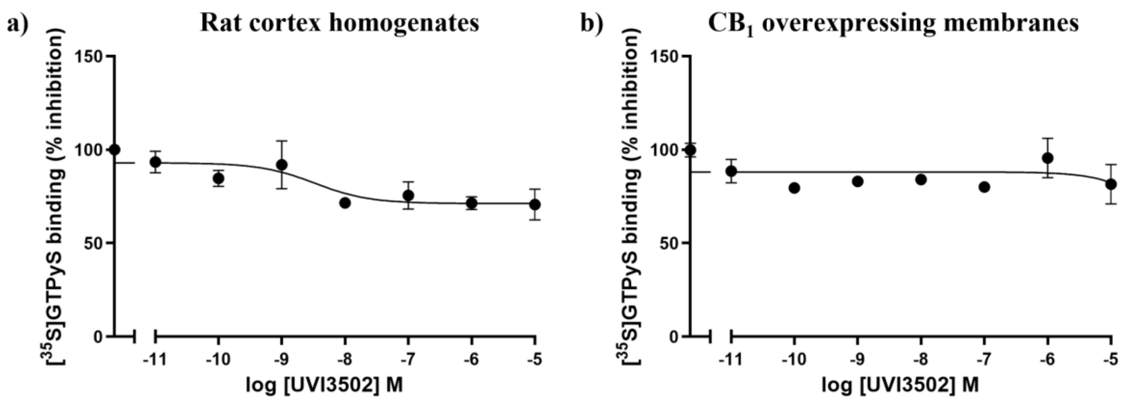

Figure S1. [^{35}S]GTPγS binding curves in the presence of UVI3502 in concentrations ranging from 10^{-11} M to 10^{-5} M in a) membrane homogenates from rat brain cortex, b) CB_1 overexpressing membranes. Note that UVI3502 does not stimulate [^{35}S]GTPγS binding, and only slightly decreases baseline levels of [^{35}S]GTPγS binding in membrane homogenates from rat brain cortex (EC_{50} 4.06 ± 3.27 nM, R^2=0.60), indicating it is acting as an antagonist. The curve represents the mean of n= 2 independent experiments.

Table S3. [^{35}S]GTPγS binding in different brain areas of rats (n = 5) and mice (n = 5) in the presence of CP55,940 (10 μM) alone, in the presence of UVI3502 (10 μM) alone and in the presence of both CP55,940 and UVI3502 (both at 10 μM).

	Stimulation (% over basal)					
	Sprague-Dawley rats			Swiss mice		
**Brain region**	**CP55,940**	**UVI3502**	**CP55,940 + UVI3502**	**CP55,940**	**UVI3502**	**CP55,940 + UVI3502**
Amygdala	49 ± 12	-35 ± 19	-47 ± 30##	88 ± 60	-16 ± 51*	0 ± 78
Cerebellum (gray matter)	68 ± 77	-16 ± 51	-46 ± 31#	128 ± 59	-43 ± 9**	6 ± 23##
Cortex	40 ± 25	-45 ± 24**	-60 ± 10##	40 ± 31	-55 ± 9**	-51 ± 11##
Globus pallidus	833 ± 352	-3 ± 46**	89 ± 42##	412 ± 189	-46 ± 29*	91 ± 104#
Hippocampus dorsal	31 ± 9	-40 ± 26*	-62 ± 6#	77 ± 23	-38 ± 12**	-36 ± 8#
Nucleus basalis magnocellularis	164 ± 35	-35 ± 30**	-40 ± 11##	91 ± 68	-20 ± 65*	-30 ± 37#
Striatum	83 ± 81	-34 ± 25*	-54 ± 23##	56 ± 36	-52 ± 18**	-52 ± 13##
Substantia nigra	314 ± 222	-41 ± 24**	14 ± 80#	747 ± 164	-58 ± 11**	181 ± 128#

CP55,940 *vs* . UVI3502 (*), CP55,940 *vs* . CP55,940 + UVI3502 (#). Kruskal–Wallis test, *post-hoc* test Dunn's multiple comparison, *# $p < 0.05$; **## $p < 0.01$.

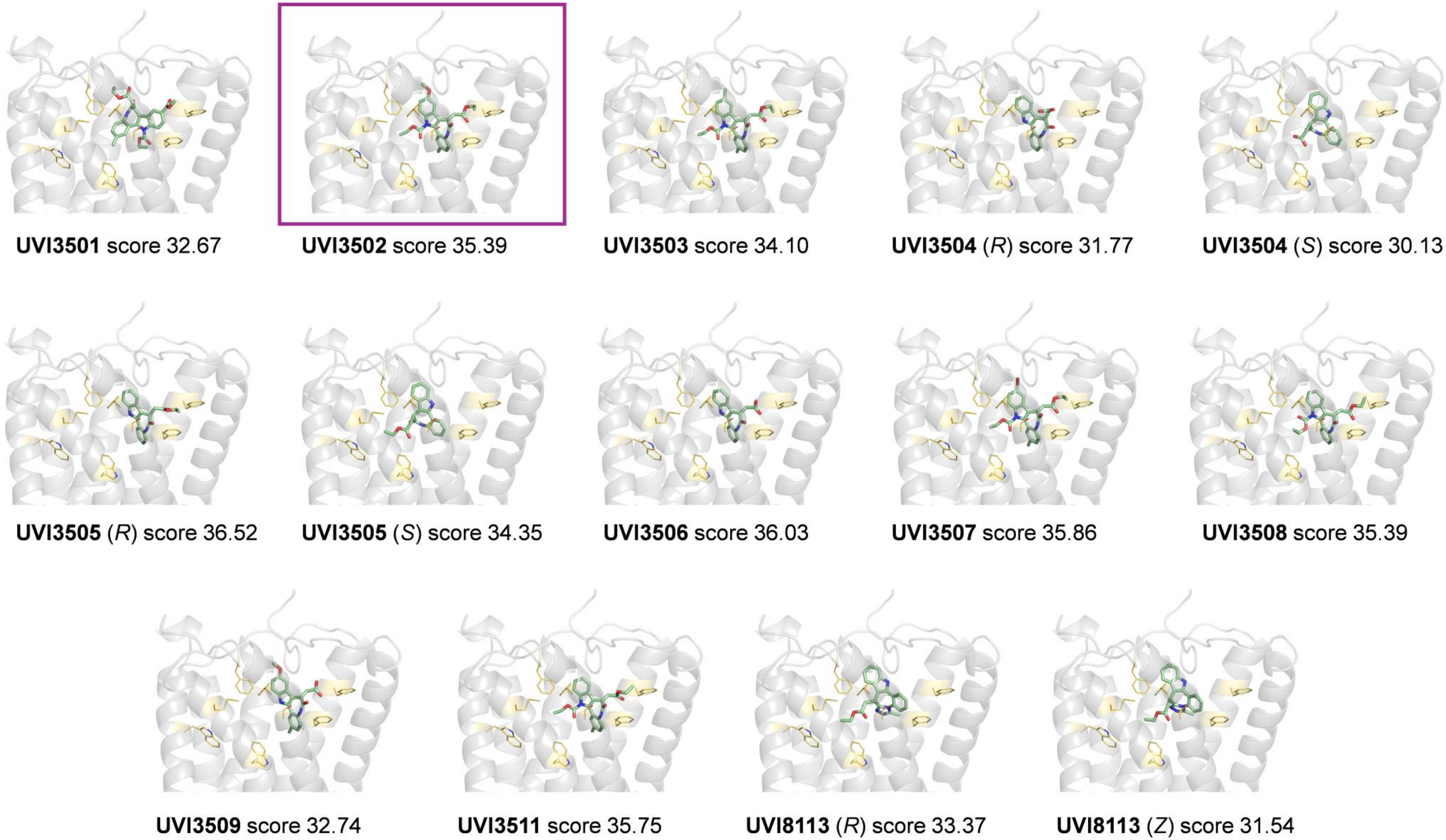

Figure S2. Docking poses of UVI3502 (highlighted with a purple box) and related analogues (in green sticks) on human cannabinoid receptor CB_1 (receptor structure taken from PDB 5TGZ). Key residues for inverse agonist binding are shown as yellow sticks.

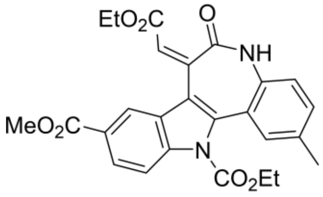

UVI3501 score 32.67

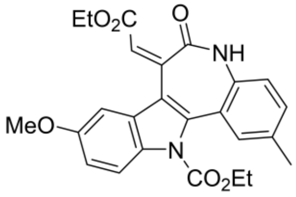

UVI3502 score 35.39

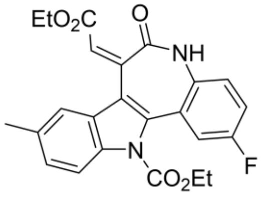

UVI3503 score 34.10

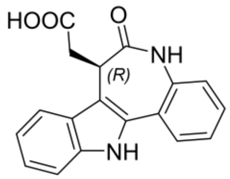

UVI3504 (*R*) score 31.77

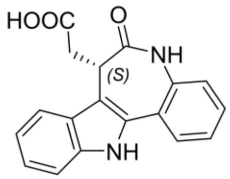

UVI3504 (*S*) score 30.13

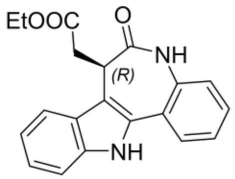

UVI3505 (*R*) score 36.52

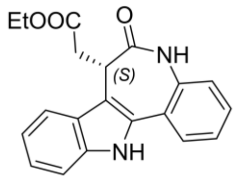

UVI3505 (*S*) score 34.35

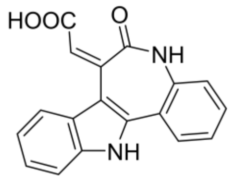

UVI3506 score 36.03

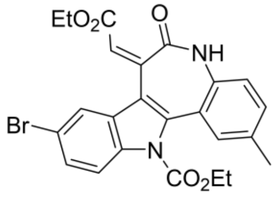

UVI3507 score 35.86

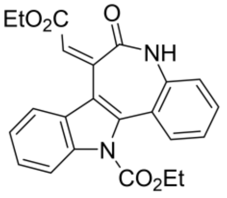

UVI3508 score 35.39

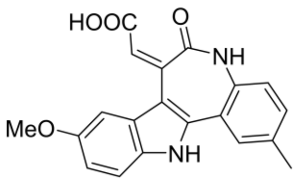

UVI3509 score 32.74

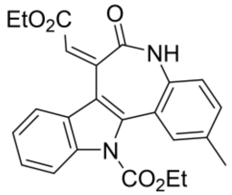

UVI3511 score 35.75

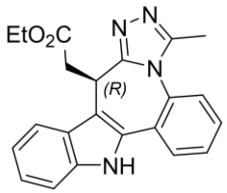

UVI8113 (*R*) score 33.37

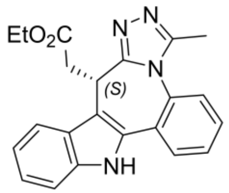

UVI8113 (*S*) score 31.54

Figure S2 (cont.). Chemical structures of UVI3502 (highlighted with a purple box) and related analogues, together with their docking scores on human cannabinoid receptor CB_1 (receptor structure taken from PDB 5TGZ).

REFERENCES

1. Denis JG, Franci G, Altucci L, Aurrecoechea JM, de Lera ÁR, Álvarez R. Synthesis of 7-alkylidene-7,12-dihydroindolo[3,2-d]benzazepine-6-(5H)-ones (7-alkylidene-paullones) by N-cyclization–oxidative Heck cascade and characterization as sirtuin modulators. *Org Biomol Chem*. 2015;13(9):2800-2810. doi:10.1039/C4OB02493A

2. Vaz B, Martínez C, Cruz F, et al. Palladium-Catalyzed Aminocyclization–Coupling Cascades: Preparation of Dehydrotryptophan Derivatives and Computational Study. *J Org Chem*. 2021;86(13):8766-8785. doi:10.1021/acs.joc.1c00636
